# Race-specific coregulatory and transcriptomic profiles associated with DNA methylation and androgen receptor in prostate cancer

**DOI:** 10.1186/s13073-024-01323-6

**Published:** 2024-04-02

**Authors:** Swathi Ramakrishnan, Eduardo Cortes-Gomez, Sarah R. Athans, Kristopher M. Attwood, Spencer R. Rosario, Se Jin Kim, Donald E. Mager, Emily G. Isenhart, Qiang Hu, Jianmin Wang, Anna Woloszynska

**Affiliations:** 1 Department of Pharmacology and Therapeutics, Roswell Park Comprehensive Cancer Center, Buffalo, NY 14263 USA; 2 Department of Bioinformatics and Biostatistics, Roswell Park Comprehensive Cancer Center, Buffalo, NY 14263 USA; 3 Department of Biostatistics, SUNY University at Buffalo, Kimball Tower, Buffalo, NY 14214 USA; 4 Department of Pharmaceutical Sciences, SUNY University at Buffalo, Buffalo, NY 14214 USA; 5 Enhanced Pharmacodynamics, LLC, Buffalo, NY 14203 USA; 6 Department of Cancer Genetics and Genomics, Roswell Park Comprehensive Cancer Center, Buffalo, NY 14263 USA

## Abstract

**Background:**

Prostate cancer is a significant health concern, particularly among African American (AA) men who exhibit higher incidence and mortality compared to European American (EA) men. Understanding the molecular mechanisms underlying these disparities is imperative for enhancing clinical management and achieving better outcomes.

**Methods:**

Employing a multi-omics approach, we analyzed prostate cancer in both AA and EA men. Using Illumina methylation arrays and RNA sequencing, we investigated DNA methylation and gene expression in tumor and non-tumor prostate tissues. Additionally, Boolean analysis was utilized to unravel complex networks contributing to racial disparities in prostate cancer.

**Results:**

When comparing tumor and adjacent non-tumor prostate tissues, we found that DNA hypermethylated regions are enriched for PRC2/H3K27me3 pathways and EZH2/SUZ12 cofactors. Olfactory/ribosomal pathways and distinct cofactors, including CTCF and KMT2A, were enriched in DNA hypomethylated regions in prostate tumors from AA men. We identified race-specific inverse associations of DNA methylation with expression of several androgen receptor (AR) associated genes, including the *GATA* family of transcription factors and *TRIM63*. This suggests that race-specific dysregulation of the AR signaling pathway exists in prostate cancer. To investigate the effect of AR inhibition on race-specific gene expression changes, we generated in-silico patient-specific prostate cancer Boolean networks. Our simulations revealed prolonged AR inhibition causes significant dysregulation of TGF-β, IDH1, and cell cycle pathways specifically in AA prostate cancer. We further quantified global gene expression changes, which revealed differential expression of genes related to microtubules, immune function, and TMPRSS2-fusion pathways, specifically in prostate tumors of AA men. Enrichment of these pathways significantly correlated with an altered risk of disease progression in a race-specific manner.

**Conclusions:**

Our study reveals unique signaling networks underlying prostate cancer biology in AA and EA men, offering potential insights for clinical management strategies tailored to specific racial groups. Targeting AR and associated pathways could be particularly beneficial in addressing the disparities observed in prostate cancer outcomes in the context of AA and EA men. Further investigation into these identified pathways may lead to the development of personalized therapeutic approaches to improve outcomes for prostate cancer patients across different racial backgrounds.

**Supplementary Information:**

The online version contains supplementary material available at 10.1186/s13073-024-01323-6.

## Background

Prostate cancer is a devastating disease, with almost 300,000 men diagnosed each year and ~12% succumbing to this disease annually [1]. African American (AA) men have the highest prostate cancer incidence and associated mortality compared to any other racial subgroup [1, 2]. Multiple factors, including socioeconomic status, access to healthcare, and environmental factors, contribute to racial disparities in prostate cancer [3, 4]. However, studies that adjusted for socioeconomic status and healthcare access show that racial disparities in prostate cancer incidence still prevail [5, 6]. Even when AA men are diagnosed at a younger age with less aggressive primary prostate cancer, defined as having a Gleason score of 6 or less, they still have an increased mortality rate [6]. These observations underscore the importance of understanding the underlying biology of primary prostate cancer associated with clinical outcomes in AA men.

Delineating molecular factors, including epigenetic alterations, that contribute to disease biology requires a thorough investigation of newly diagnosed and therapy naïve prostate cancer from AA men. Increased promoter DNA methylation at *GSTP1*, *PRDM13*, and *RAR-β2*, as well as other genes, are frequently found in prostate tumors from EA and AA men [7–9]. Unsupervised hierarchical cluster analyses show that DNA hypermethylation patterns at CpG islands, which include transcription start sites, are similar between different regions from the same prostate tumor [10]. In advanced prostate cancer, DNA methylation patterns are similar between tumors obtained from different metastatic sites from the same individual [11]. These observations suggest that DNA methylation events maintain intratumoral, intraindividual, and clonal stability in prostate cancer. These studies have established DNA methylation as an attractive clinical biomarker; however, most of these biomarkers are derived from clinical samples solely obtained from EA men. In prostate cancer from AA men, DNA methylation alterations at specific loci, including *ESR2* and *FGF6*, are associated with higher Gleason Score tumors [12]. DNA methylation at *NKX2-5* also correlates to AA ancestry in prostate tumors and adjacent non-tumor tissues [7]. These previous contributions demonstrate the importance of differential DNA methylation patterns in prostate cancer from AA men.

Alterations to the epigenome are important, given DNA methylation regulates gene expression by cooperating with other epigenetic modifying enzymes, such as EZH2, that can activate or repress gene transcription [13]. DNA methylation patterns can also dictate transcription factor binding; for example, the pioneer transcription factor GATA4 binds preferentially to methylated CpG sites [14]. Whether the interaction between DNA methylation and associated proteins is distinct between prostate cancer of AA and EA men is not known. Understanding these interactions will help establish how epigenetic processes relate to altered transcriptional programs fundamental to prostate cancer biology in AA men. Consequently, it will uncover actionable biology to reduce racial disparities in prostate cancer.

Epigenetic-induced gene expression changes can result in altered downstream signaling; however, these changes are often pleiotropic with impacts on multiple pathways in a given cell and heterogenous tumor. A novel approach to study crosstalk between known signaling pathways in prostate cancer, which also accounts for mutations and copy number alterations, is the utilization of patient-specific Boolean networks [15]. These integrated platforms have never been utilized to delineate prostate cancer biology in the context of race. Disease biology and response to therapy are naturally interconnected biochemical networks giving rise to complex and dynamic clinical phenotypes [16]. Therefore, a systems pharmacology approach is uniquely suitable for investigating race-specific biological differences in primary therapy naïve tissues, and assessing how they may differentially respond to standard-of-care therapies.

A significant hindrance in identifying molecular determinants of prostate cancer racial disparities comes from the limited number of samples and studies utilizing multi-omics approaches to directly compare primary tumors from AA and EA men. In the current study, we intentionally included primary prostate cancer and adjacent non-tumor tissues from AA men and performed a comparative analysis with similar samples obtained from EA men. Although we observed similar DNA methylation patterns between prostate tumors and adjacent non-tumor tissues, these patterns are enriched for distinct chromatin-modifying enzymes. These included EZH2 in DNA hypermethylated regions of prostate tumors in AA and EA men and CTCF in hypomethylated regions of only prostate tumors in AA men. Utilization of our patient-specific prostate cancer Boolean networks revealed the potential role of prolonged AR therapy in inducing gene expression changes in TGF-β, IDH1, and cell cycle pathways specifically in prostate cancer of AA men. Furthermore, downregulation of a subset of immune-associated pathways in prostate tumors was associated with reduced risk of disease progression only in AA men. Overall, our study begins to dissect race-specific DNA methylation and AR-mediated transcriptional alterations that may underlie prostate cancer biology and are associated with clinical outcomes.

## Methods

### Cohorts analyzed in study

All clinical samples were collected with informed consent between 2005 and 2017, before any treatment and at the time of radical prostatectomy at Roswell Park. All samples were de-identified before analyses. Patient demographic and clinical characteristics were reported by self-identified race and compared using the mean, median, standard deviation, and range for continuous variables, and by using frequencies and relative frequencies for categorical variables (Additional File 1, Table S1). Comparisons were made using the Mann-Whitney *U* and Fisher’s exact tests, as appropriate.

Samples from a total of 41 EA patients and 35 AA patients were collected for analysis. DNA methylation was analyzed via methylation array (described in subsequent sections) on 37 tumor (T) and 37 non-tumor (NT) samples from EA patients, and 32 T and 30 NT samples from AA patients. RNA sequencing was performed on 31 T and 27 NT samples from AA patients, and 32 T and 31 NT samples from AA patients. Between these two cohorts, these was an overlap comprised of NT = 27 and T = 31 AA patient samples and NT = 31, T = 32 EA patient samples. This overlapping cohort was analyzed via multi-omics analysis (described in subsequent sections), and characteristics are described in Additional File 1, Table S2. *N* = 15 patient samples (AA: 7, EA: 8, all T) were further profiled via whole-exome sequencing. This cohort (n=15) was utilized for Boolean network generation and modeling of AR inhibition (described in subsequent sections). Tissue microarray (TMA) analysis of AR expression was performed on prostate tumor tissues and adjacent non-tumor tissues from AA (T: 107, NT: 107) and EA men (T: 133, NT: 133) (TMA demographics described in Supplementary File 1, Table S3). 20 AA T samples from the TMA overlap with the cohort used for RNA sequencing. There is no overlap between EA TMA and sequencing samples.

### DNA methylation and DMR annotation

DNA extraction was performed using a Qiagen Qiamp DNA mini kit (Cat#51304). 10 EA (non-tumor (NT) = 5, tumor (T) = 5) and 62 AA (NT = 30, T = 32) samples were analyzed by the Illumina Infinium Human Methylation450 BeadChip DNA methylation array (subsequently referred to as 450K array), which includes over 450,000 methylation sites per sample. Additional EA (NT = 32, T = 32) samples were analyzed by the Ilumina Infinium MethylationEPIC array, which includes over 850,000 methylation sites and covers over 90% of the 450K array content (Additional File 2, Fig. S1A). Briefly, the Infinium methylation protocol combines bisulfite conversion of genomic DNA and whole genome amplification with direct, array-based capture and scoring on the CpG loci. First, DNA samples are quantitated and 500 ng DNA are plated in 96-well plates as per layout suitable for 8-sample EPIC chip format. The DNA samples are treated with sodium bisulfite using an Illumina-specific bisulfite conversation kit (Zymo EZ DNA methylation kit). Subsequently, DNA samples are isothermally amplified in an overnight step to increase the amount of DNA. Whole genome amplified DNA samples are then fragmented, precipitated, resuspended, and hybridized to EPIC BeadChip. The chips are incubated overnight for allele-specific hybridization and washed the next day to remove unhybridized and nonspecifically hybridized DNA. Following the wash, chips undergo single base extension and staining. Two bead types correspond to each CpG locus, one bead type corresponds to methylated (C) another bead type to the unmethylated (T) site. Lastly, the chips are scanned using the Illumina iScan system, using a laser to excite the fluorophore of the single-base extension product on the beads. The scanner records high-resolution images of the light emitted from the fluorophores. The raw image data is processed using Illumina’s GenomeStudio V2011.1. Minfi (v1.38), a methylation array Bioconductor package, was used to read raw intensities, preprocess, and normalize values from methylation arrays [17]. Probes with detection *P*-values >0.01 were filtered out before preprocessing. PreprocessFunnorm was used to normalize individual arrays. The combineArrays function was used to merge data from the 450K and EPIC arrays so that only the probes that overlap between the 450K and EPIC arrays were used for further analyses (total 449,636 probes, Additional File 2, Fig. S1A). *M* value was calculated as *M* = log2((*M*+*a*)/(*U*+*a*)). M is methylated intensity, U is unmethylated intensity, and a is a constant offset (by default, *a* = 100). The comBat function from sva (v3.40.0), a Bioconductor package [18], was used for batch-correction of *M*-values (Additional File 2, Figs. S1B and S1C). DMRcate (v2.12), an R package, [19] was used to annotate and establish differentially methylated regions (DMRs) between tumors and adjacent non-tumor tissues as well as race-specific differences in DMRs. DMRs were ranked by Fisher’s multiple comparison statistic based on maximum differences (maxdiff) between CpG probes in a given DMR and mean differences (meandiff) that calculate the average of differences between each CpG probe in a given DMR. A Fisher statistic of *P*<0.05 was considered to be significant. The rmSNPandCH function from DMRcate was used on the overlapping sites to remove CpGs within 2bp of a known SNP. We ran the LISA cistrome pipeline [20] on identified DMRs to infer enrichment of chromatin remodeling enzymes and transcription factor motifs. DNA methylation array datasets are deposited into NCBI GEO, accession numbers GSE262522 and GSE262524.

### DMR pathway enrichment

DMRs identified by maxdiff values were used for enrichment analysis. DMRs with opposing maxdiff and meandiff values were excluded. Gene sets were acquired from the Molecular Signatures Database (MSigDB) [21], which included curated gene sets (C2), ontology gene sets (C5), oncogenic signature gene sets (C6), and hallmark gene sets (H). Gene set enrichment analysis of DMRs was then performed using the leading-edge gene for each region and maxdiff as the test statistic with the fgsea R package.

### Manhattan Plots for DMRs

The median location for each DMR was calculated and plotted on the *x*-axis of sequential base pairs along chromosomes. Hypermethylated DMRs were plotted as −log10(FDR) on the *y*-axis, while hypomethylated DMRs were plotted as log10(FDR) on the *y*-axis. This allowed for the visual separation of hyper- and hypo- methylated DMRs. The top ten hyper- and hypo- methylated DMRs were labeled using the leading-edge gene for each region where applicable, or the DMR identified from the analysis. Dashed lines at +/− log10(.05) and +/− log10(5e−8) represent individual DMR significance and genome-wide significance, respectively.

### RNA sequencing

Paired-end RNA sequencing was performed on RNA isolated from clinical samples (AA: NT = 27, T = 31, EA: NT = 31, T = 32). RNA extraction was performed using a Qiagen miRNeasy mini kit (Cat#217044). Briefly, 500 ng total RNA was used to prepare the sequencing libraries with the Illumina TruSeq Stranded Total RNA Library Prep Kit with Ribo-Zero Gold (Illumina Inc, Cat. No. RS-122-2301). Following the manufacturer’s instructions, rRNA was depleted from total RNA, and the remaining RNA was purified, fragmented, and primed for cDNA synthesis. Fragmented RNA was reverse-transcribed into first-strand cDNA using random primers. The RNA template was removed, and a replacement strand was synthesized by incorporating dUTP in place of dTTP to generate double-stranded (ds) cDNA. AMPure XP beads (Beckman Coulter) were used to separate the ds cDNA from the second strand reaction mix resulting in blunt-ended cDNA. A single “A” nucleotide was added to the 3′ ends of the blunt fragments. Multiple indexing adapters, containing a single “T” nucleotide on the 3′ end of the adapter, were ligated to the ends of the ds cDNA and prepared for hybridization onto a flow cell. Adapter ligated libraries were amplified by PCR, purified using Ampure XP beads, and validated for appropriate size on a 4200 TapeStation D1000 Screentape (Agilent Technologies, Inc.). The DNA libraries were quantitated using the KAPA Biosystems qPCR kit and pooled together in an equimolar fashion, following experimental design criteria. Each pool was denatured and diluted to 2.4 pM with 1% PhiX control library added. The resulting pool was loaded into a 150-cycle NextSeq High Output Reagent cartridge for 75-cycle paired-end sequencing and run on a NextSeq500 following the manufacturer’s recommended protocol (Illumina Inc.).

The raw reads that passed the quality filter from Illumina RTA were first preprocessed using FastQC (v0.10.1) [22] for sequencing base quality control to be mapped to the human reference genome GrCh38 and corresponding RefSeq annotation database using TopHat (v2.0.13) [23]. A second round of quality control using RSeQC (v2.6.5) [24] was applied to mapped bam files to identify potential RNA-seq library preparation problems. From the mapping results, read counts for genes were obtained by HTSeq [25] using the intersection-strict option. Differentially expressed (DE) genes were identified using DESeq2 (v1.38.3) [26]. Differential expression rank order was utilized for subsequent Gene Set Enrichment Analysis (GSEA), performed using the clusterProfiler package in R. Gene sets queried included the Hallmark, Canonical pathways, and GO Biological Processes Ontology collections available through the Molecular Signatures Database (MSigDB) [21].

### Multi-omics integration analyses

Maxdiff (from the methylation array data) and log2(Fold Change) (log2FC) (from the RNAseq expression data) statistics were utilized to quantify effect sizes (AA: NT = 27, T = 31, EA: NT = 31, T = 32). We used only the clinical specimens that were analyzed by both RNA sequencing and methylation array for multi-omics integration analyses. Clinical characteristics for this overlapping patient cohort are summarized in Additional File 1, Table S2. Gene symbols from the expression data were matched with their corresponding counterparts overlapping probe symbol(s) within a DMR. Since each DMR consists of many probes, the one with the largest probe-wise beta value range was picked as a representative for a given DMR. Sample *β*-values from that representative probe were extracted and matched with the corresponding sample DESeq2-normalized expression value. *β*-values are calculated as *β* = *M*/(*M*+*U*+*a*). M is methylated intensity, *U* is unmethylated intensity, and a is a constant offset (by default, *a* = 100). Spearman correlation was utilized to analyze THE relationship between DNA methylation and RNA sequencing.

### Roswell data formatting for Boolean network modeling

DNA was prepared for sequencing from fresh frozen samples via a Qiagen Qiamp DNA mini kit (Cat#51304) for *n* = 15 patient samples (AA: 7, EA: 8). Only tumor samples with purity above 70% tumor nuclei content were used for analysis. We initiated our process with high-quality paired-end reads from whole exome sequencing that passed the Illumina RTA filter. These reads were aligned against the NCBI human reference genome (GRCh37) utilizing a suite of bioinformatics tools, including BWA [27] and Samtools [28]. Multiple variant detection tools, including Mutect2 [29], VarScan2 [30], VarDict [31], SomaticSniper [32], MuSE [33], and Strelka2 [34], were employed to identify potential single nucleotide variants (SNVs) and insertions and deletions (indels). The major-vote consensus mode by SomaticSeq [35] was used to further filter all putative somatic variants. Finally, we annotated all identified mutations using the Variant Effect Predictor (VEP) [36], leveraging its comprehensive collection of genomic annotations sourced from the Ensembl database. All analyses were conducted following best practices and utilizing reproducible pipelines facilitated by the RcwlPipelines package [37]. SNPs were organized in a matrix with columns indicating usual genomic locations, features, SNP type and dbSNP identifier followed by Polyphen and SIFT annotations.

Allele-specific somatic copy number variations (CNVs) were identified utilizing FACETS (v0.6.2) [38]. By extracting reference and variant allele read counts from the bam file for the polymorphic sites present in the dbSNP database, FACETS performed a joint segmentation analysis on both total and allelic copy ratios. Results include genomic ranges and log-ratio (lRR), values which are broken down to determine per sample ploidy (−2: lRR ≤ − 1.1, − 1: − 1.1< lRR ≤ − 0.2, 0: − 0.2 < lRR ≤ 0.2, 1: 0.2 < lRR ≤ 0.7, 2: lRR > 0.7 ). Each range is assigned to resulting overlapping genes using GRCh37 refSeq annotation. Data were transformed by creating an incidence matrix using ploidy values with the gene annotation symbols as rows and columns with the total number of samples.

For gene expression, raw counts and normalized data were formatted as matrices where rows are gene symbols and columns are samples.

### AR inhibition in prostate cancer Boolean network

Fifteen subject-specific Boolean network models of prostate cancer signaling were generated for the Roswell Park cohort through methods developed by Béal et al. [39]. 313 subject-specific Boolean network models of prostate cancer signaling were extracted from a prior analysis that utilized patient data from The Cancer Genome Atlas [15]. The structural network is comprised of 133 nodes in which each node represents a gene or protein and is associated with discrete levels of activity (0 or 1). Four hundred ninety-nine edges connect the nodes, each representing a regulatory interaction (positive or negative) between the source and target nodes [40]. Subject-specificity is defined by the model parameters obtained from discrete data, such as mutations and copy number alterations, and continuous data, such as RNA sequencing. The models were perturbed to mimic drug-specific effects, and each patient model was simulated to steady state to capture the node’s activity over an arbitrary unit of time. In this study, the androgen receptor (AR) node activity was fixed to a discrete value of 0 to mimic the effect of an AR inhibition or pharmacological antagonism, and the individual subject-specific models were simulated to steady state. The changes in the area under the curve (AUC) of each node before and after perturbation were calculated according to the following equation:

$$\Delta \textrm{AUC}\ \left(\%\right)=\frac{{\textrm{AUC}}_{ap}-{\textrm{AUC}}_{bp}}{{\textrm{AUC}}_{bp}}\times 100$$

with *bp* and *ap* subscripts denoting before and after perturbation, and values were compared between AA and EA men. Network nodes were grouped according to their association with relevant signaling pathways in prostate cancer biology. Differences in the change of AUC values after AR inhibition between AA and EA men were tested for statistical significance using the Mann-Whitney *U* test adjusted multiple comparisons using the Benjamini-Hochberg procedure if the change in AUC values were ≥ 3% [41]. An alpha value of 0.05 was considered statistically significant.

### Immunohistochemistry, Image J quantitation, and statistical analyses

Tissue microarrays (AA, *n* = 107; EA, *n* = 133) were stained with an AR antibody (Agilent, #M3562) and digitally scanned using an Aperio Scanscope. Individual prostate cancer images were captured with Scanscope, and the ImmunoRatio plugin (ImageJ, NIH) was used to analyze the percentage of AR-positive nuclei. For the percent of AR-positive nuclei, the association with each patient characteristic was evaluated using the one-way analysis of variance (ANOVA) model. The percent of AR-positive nuclei was then modeled as a function of each patient characteristic, race, or age and their interaction using a general linear model. *F*-tests about the appropriate linear combination of model estimates were used to evaluate: (A) the association between the patient characteristic and AR within each race or age group; and (B) the racial or age effect on the association between AR and the patient characteristic (i.e., by testing the interaction term). All analyses were conducted in SAS v9.4 (Cary, NC) at a significance level of *P* < 0.05.

### GSVA scoring and HR calculations

Gene Set Variation Analysis (GSVA) scoring was performed with the GSVA R package [42]. GSVA scores were built on single sample GSEA by using a non-parametrical approach and Kolmogorov–Smirnov (KS)-like random walk statistic after normalizing the gene expression profiles with appropriate kernel estimation functions depending on whether expression levels were continuous or counts data. Enrichment scores compared the overall expression of two samples within the same gene set. Given a gene set, samples with positive enrichment values had more genes at the top-rank expression levels than samples whose genes had lower levels (for example, the bottom of the ranked expression list) with respect to the gene set. RNA-seq dataset is deposited into NCBI GEO, accession number GSE237995 [43].

Progression-free survival (PFS) was defined as the time from radical prostatectomy until persistent disease, recurrence, or last follow-up. For patients with persistent disease, the PFS was calculated as 1 day. Persistent disease after radical prostatectomy for our purposes was defined as follows: 1. PSA levels don’t fall to undetectable levels after surgery (> 0.03 to < 0.2 ng/mL) and is associated with adverse pathological factors (stage T3a or above, diffusely positive surgical margins) and 2. PSA levels > 0.2 ng/mL.

Hazard ratio (HR) estimation via Cox proportional hazards model regression analyses were carried out to examine the association with PFS across gene expression and GSVA biological pathway scores. This semi-parametric model regression model allows to study the relationship between survival endpoints (this case PFS) and other predictor variables of interest. The model’s coefficients inform about direction and magnitude of the covariate effects on the hazard function. Positive coefficient values indicate increased hazard of the event to occur, while negative values can be interpreted as ‘protection’ or decreased hazard of the event occurring. Three separate models were fitted to estimate HR scores for all samples and individually for each race. The Benjamini-Hochberg’s method was used to account for multiple testing and identify significant genes (adjusted *P*-value < 0.1). Effect sizes were reported using HR.

## Results

### Hypomethylated regions of DNA are enriched for chromatin remodelers in a race-specific manner

**Fig. 1:**
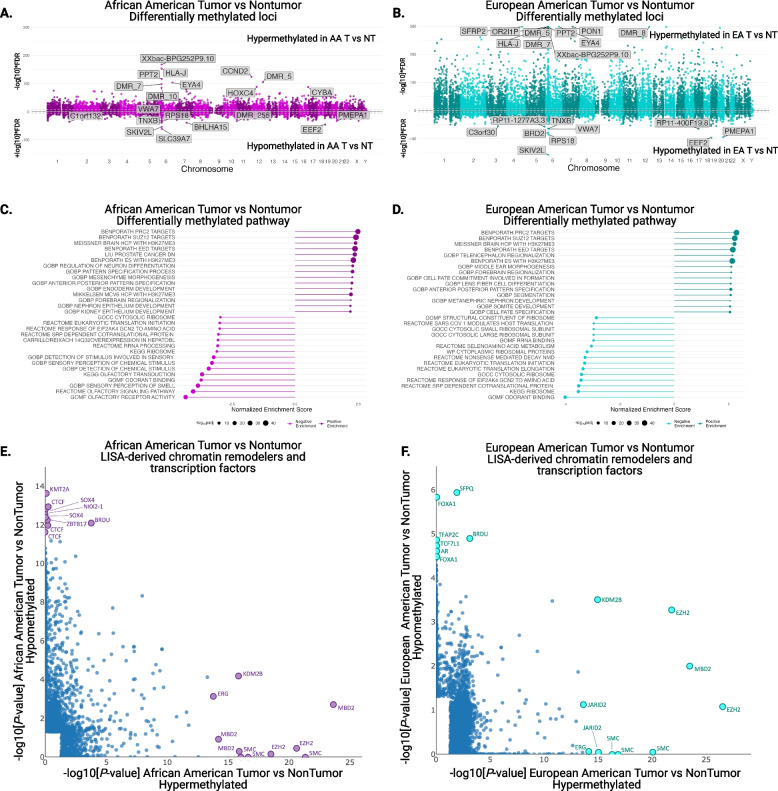
Differentially methylated regions between prostate tumors from AA and EA men reveal race-specific enrichment of pathways and transcript factors. **A** and **B** Manhattan plots representing differentially methylated regions in prostate tumors from AA (**A**) and EA men (**B**) across all chromosome locations. HyperDMRs are in the top half, represented as −log[10]FDR and hypoDMRs are in the bottom half, represented as log[10]FDR. **C** and **D** Enriched pathways associated with DMRs in prostate tumors from AA (**C**) and EA (**D**) men. **E** and **F** Lisa-derived enrichment of epigenetic regulators enriched in prostate tumors from AA (**E**) and EA men (**F**). *X-axis:* represents hypermethylated regions in prostate tumors, and *Y-axis*: represents hypomethylated regions in prostate tumors

We examined primary, therapy naïve tumor and adjacent non-tumor tissues from AA (*n* = 31 T, 27 NT) and EA men (*n* = 32 T and 32 NT) to determine DNA methylation levels in prostate cancer (demographics in Additional File 1, Table S1). Principal component analysis (PCA) showed that DNA methylation, measured as *β*-value for each CG site (described in the “Methods” section), distinguishes prostate tissues between AA and EA men (Additional File 2, Fig. S2A), based on PC1 and PC2. A PERMANOVA test (10,000 permutations) confirms that tumor samples are significantly different between races (*R*^2^= 0.135, *p*val < 0.0001). PCA did not clearly differentiate between tumor and adjacent non-tumor tissues based on DNA methylation patterns (Additional File 2, Fig. S2A). As a first step to investigate tumor-specific changes in DNA methylation, we established differentially methylated regions (DMRs) using “DMRcate,” an R package [19]. We identified DMRs between tumor and adjacent non-tumor tissues within each race. The top 1000 DMRs, from both race- and tissue- comparisons, are represented in Additional File 2, Fig. S2B, and all DMRs are listed in Additional File 3. An increase in DNA methylation in tumors compared with adjacent non-tumor tissue is referred to as hyperDMRs (top half, represented as −log[10]FDR, in Fig. 1A–B; Additional File 2, Fig. S3A), and a decrease in methylation in tumors compared with adjacent non-tumors is referred to as hypoDMRs (bottom half, represented as log[10]FDR, in Fig. 1A–B; Additional File 2, Fig. S3A). The number of significant hyperDMRs was higher than hypoDMRs in prostate tumors (AA: HypoDMR = 6280/HyperDMR = 11,866 Fig. 1A; EA: HypoDMR = 7715/HyperDMR = 22,042 Fig. 1B). The number of hyperDMRs was twice as low in AA men compared to EA men. These observations, in addition to the PCA plots and lower number of hyperDMRs, suggest that DNA methylation between tumors and adjacent non-tumor tissues is more similar in AA men than EA men. Conversely, we found that adjacent non-tumor tissues from AA men have increased DNA methylation at specific gene regions compared to EA men (red gradient vs blue gradient in adjacent non-tumor tissues, purple rectangles in Additional File 2, Fig. S2B).

We assigned DMRs to specific genes based on location and then performed gene set enrichment analysis (GSEA) on these DMR-associated genes to identify specific pathways that were enriched between comparison groups. HyperDMRs were enriched in the PRC2 and H3K27me3 pathways in a race-independent manner (Fig. 1C, D). The association of hyperDMRs with PRC2/H3K27me3-associated pathways was further supported by LISA-based (epigenetic Landscape In Silico deletion Analysis) results. The LISA-based computational algorithm uses ChIP-derived histone marks and chromatin accessibility profiles from Cistrome databases to predict cis-regulatory elements that can regulate gene expression in a given dataset [20]. We applied LISA to the annotated DMRs (AA (*n* = 31 T, 27 NT) and EA (*n* = 32 T and 32 NT)) to identify potential transcription factors or co-regulators of gene expression. LISA-derived prediction showed EZH2 enrichment in hypermethylated regions independently of race (Fig. 1E, F). The PRC2 complex primarily methylates histone 3 lysine 27 (H3K27) to repress gene transcription [44]. These observations suggest that the PRC2 complex is present in hypermethylated regions of prostate tumors and can potentially suppress gene transcription. HypoDMRs were enriched in the olfactory and ribosomal pathways in a race-independent manner (Fig. 1C, D). LISA-derived prediction showed enrichment of chromatin-remodeling enzymes, including CTCF and KMT2A, at hypoDMRs of prostate tumors from AA men but not EA men (Fig. 1E, F). HypoDMRs in prostate tumors of EA men were enriched for previously known prostate cancer-associated transcription factors, including FOXA1 and AR [45]. These data support the conclusion that there are interactions between DNA and known prostate cancer-associated transcription factors and chromatin-remodeling enzymes. These interactions may depend on the methylation status of specific genomic regions and are distinct in prostate cancer from AA and EA men.

We also determined race- and tumor-specific changes in DNA methylation using a two-way ANOVA, to account for both race and source tissue (tumor or adjacent non-tumor). This statistical test first determines the methylation difference at each DMR between tumors and adjacent non-tumors within each race, followed by comparisons between AA and EA men (EA(T-NT)-AA(T-NT)). This allowed us to understand the magnitude of DNA methylation change in prostate tumors in relation to adjacent non-tumor tissue, in the context of race. A single DMR annotated to EIF1AY, located on the Y-chromosome, had a greater magnitude of increased methylation in prostate tumors of AA men (*P* < 0.05, represented as a dot below the second dashed line, Additional File 2, Fig. S3A). This was not surprising as the number of DMRs was much lower in AA men. The positive value resulting from differences in all the other DMRs suggested that prostate tumors from AA men have decreased methylation at specific regions of DNA compared to EA men (gray rectangles Additional File 2, Fig. S2B). These DMRs were annotated to genes crucial for the development of the nervous system, including ZIC1 and EBP41L3 (Additional File 2, Fig. S3A). In agreement with our earlier observations, LISA-derived prediction showed that decreased DNA methylation in prostate cancer was associated with distinct chromatin remodeling enzymes, including BACH1 and PCGF6, in AA men (Additional File 2, Fig. S3B). Our observations suggest that altered DNA methylation, specifically hypomethylated regions, is associated with distinct chromatin-remodeling enzymes in AA men compared to EA men.

### Differentially methylated regions in AR target genes and the GATA family of transcription factors are associated with gene expression in a race-specific manner

We investigated if differential DNA methylation correlates with race-specific changes in gene expression in prostate cancer. We calculated correlation estimates between DMRs and mRNA expression across the entire transcriptome in samples analyzed by both methylation arrays and bulk-RNA sequencing (AA: T = 31, NT = 27, EA: T = 32, *N* = 31). This allowed us to calculate and compare correlation estimates (*ρ*) between DNA methylation and gene expression within matched tumor and adjacent non-tumor tissue. We observed positive and negative correlations between DNA methylation and gene expression in tumor versus non-tumor tissues (Additional File 2, Figs. S4A–B, all correlations are listed in Additional File 4). Positive correlation between DNA methylation and gene expression has been established in other models [46]. To our knowledge, our study is the first to report an inverse correlation between *Alpha-Methylacyl-CoA Racemase* (*AMACR*) expression and methylation of *AMACR*-associated DMRs in prostate cancer and adjacent non-tumor tissues (AA: correlation estimate for tumor: − 0.62, *P*-value: < 0.001; EA: correlation estimate for tumor: − 0.68, *P*-value: < 0.001; Additional File 2, Figs. S4C–D; Additional File 4). AMACR is a verified biomarker overexpressed in prostate cancer [47]. Compared to adjacent non-tumor tissue, *AMACR* is overexpressed in prostate tumors from AA men (log2fold change: 1.85, *P* < 0.001) and EA men (log2fold change: 2.70, *P* < 0.001) (Additional File 5). Our observations suggest a potential mechanistic link between DNA hypomethylation and *AMACR* gene expression in prostate cancer.

**Fig. 2:**
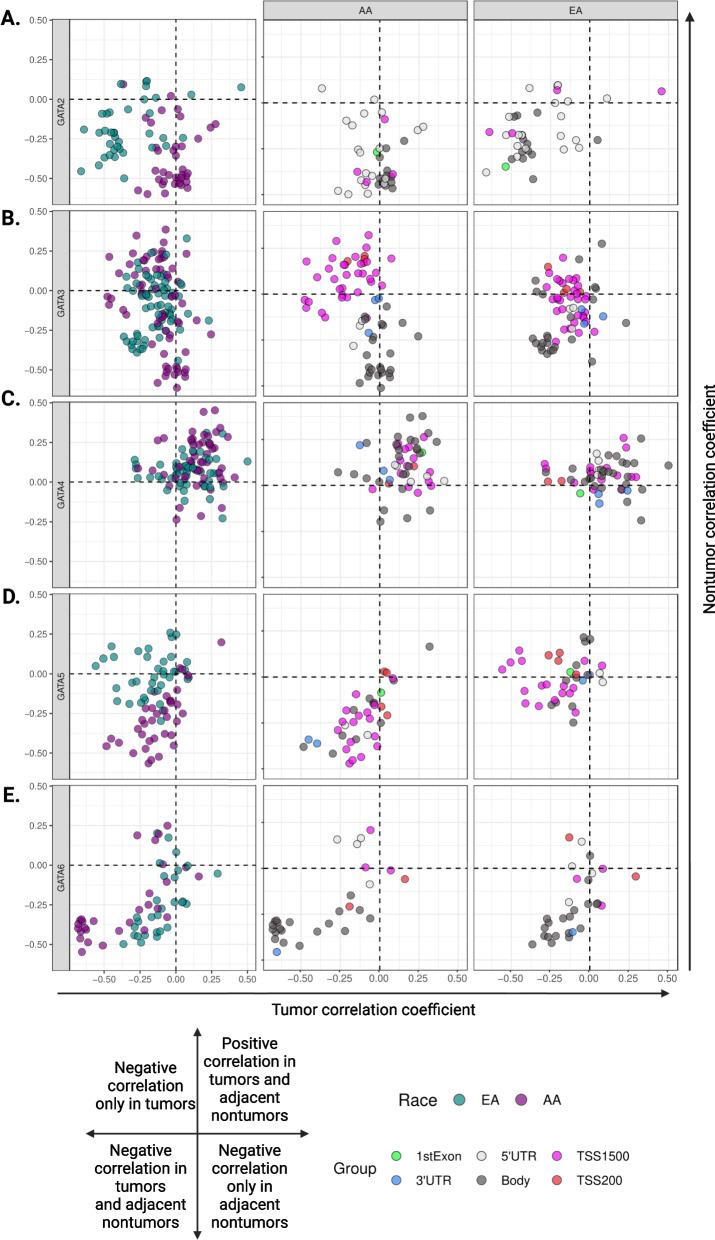
GATA transcription factor DNA methylation and gene expression are associated in a race-specific manner. Each dot represents *ρ*, correlation coefficient, between methylation and gene expression. *Top left quadrant* represents a negative correlation between DNA methylation and RNA expression in prostate tumors compared to adjacent non-tumor tissues; i.e., a negative correlation is only found in prostate tumors. *Left panels:* represent correlation in all prostate tumors. Purple dots represent AA men, and dark cyan dots represent EA men. *Middle panels:* represent annotated CpG sites in AA men. *Right panels:* represent annotated CpG in EA men

To identify race-specific associations, we focused on gene expression and DMR correlations that are distinct between AA and EA men. In prostate cancer from AA men, genes including known AR target genes *TRIM63*, *ATP2A1*, and *ARHGAP28* [48–50], showed a significant inverse correlation between gene expression and DNA methylation (Additional File 4). Additionally, we observed race-specific correlations between DNA methylation and gene expression of GATA family members (Fig. 2A–E, Additional File 6). *GATA2* and *GATA3* are known AR co-regulators in prostate cancer but any race-specific association is unknown [51]. There is also limited knowledge about the role of the other members of the GATA family in prostate cancer [52, 53]. Therefore, we investigated the association between DNA methylation and gene expression of all GATA family members.

Regardless of race, *GATA3* was significantly downregulated in prostate tumors compared to adjacent non-tumor tissues (Additional File 2, Fig. S4E, *P* < 0.001). *GATA4* was the only member of the family that showed a positive correlation between gene expression and DNA methylation at specific loci in both AA and EA patients and tumor and adjacent non-tumor tissues (Fig. 2C, *left panel*: right top quadrant, Additional File 6). Conversely, *GATA5* and *GATA6* gene expression were negatively correlated with DNA methylation at specific loci in prostate tumors and adjacent non-tumor tissues regardless of race (Fig. 2D–E, *left panel*: left bottom quadrant, Additional File 6).

We identified race-specific differences. There was a significant negative correlation between DNA methylation of the *GATA5* transcription start site and RNA expression in tumor tissue of EA men (Fig. 2D, *middle and right panels*: left top quadrant, Additional File 6). *GATA2* expression was negatively correlated with DNA methylation at specific loci in adjacent non-tumor tissues of AA men, suggesting that DNA methylation can regulate *GATA2* expression (Fig. 2A, *left panel*: left bottom quadrant, dots represent correlation coefficient estimate for individual clinical samples, Additional File 6). *GATA2* expression inversely correlated with DNA methylation at the multiple CG sites in the 5′-UTR region (*P*-value: < 0.001) in adjacent non-tumor tissues from AA men (Fig. 2A, *middle and right panels*: left bottom quadrant, Additional File 6). This suggests that DNA methylation at the 5′-UTR can inhibit transcription and therefore reduce *GATA2* gene expression. *GATA3* expression negatively correlated with DNA methylation at specific loci in a subset of prostate tumors from AA men (Fig. 2B, *left panel*: left top quadrant, Additional File 6). Significant correlations of gene expression with DNA methylation were observed explicitly at multiple CG sites at the transcription start site (*P* < 0.01) in prostate tumors from AA men (Fig. 2B, *middle and right panels*: left top quadrant, Additional File 6). Additionally, we observed an inverse correlation between DNA methylation and gene expression in annotated *GATA5* transcription start sites in adjacent non-tumor tissues from AA men (Fig. 2D, *middle and right panels*: left bottom quadrant, Additional File 6). Compared to adjacent non-tumor tissues, *GATA4* was significantly upregulated in prostate tumors from AA men (Additional File 2, Fig, S3E, *P* < 0.05). *GATA5* expression was significantly downregulated in adjacent non-tumor tissues from AA men compared to EA men (Additional File 2, Fig. S4E). *GATA5* was further downregulated in prostate tumors compared to adjacent non-tumor tissues of AA men (Additional File 2, Fig. S4E, *P* < 0.001). Our findings indicate that epigenetic regulation is an important process, which may impact race-specific expression of genes associated with AR signaling in prostate cancer and adjacent non-tumor tissues.

### Patient-specific prostate cancer Boolean network modeling reveals race-specific differences in TGF-β, IDH1, and cell cycle pathways

To understand whether differential expression of AR-associated genes (*TRIM63*, *ATP2A1*, *ARHGAP28*, and *GATA*) indicates dysregulation of AR signaling in general, we first determined AR protein expression in tissue microarrays (TMAs) consisting of prostate tumor tissues and adjacent non-tumor tissues from AA (T: 107, NT: 107) and EA men (T: 133, NT: 133). Given that nuclear AR protein drives transcriptional activity that is important for prostate cancer proliferation and survival [54], we assessed nuclear AR expression. We found race- and tissue-specific correlations between the percent of AR-positive nuclei and clinical parameters, including Gleason score and progression-free survival (Additional File 1, Table S3–4; Additional File 2, Figs. S5A–B). The percent of AR-positive nuclei was higher in adjacent non-tumor but not tumor tissues from AA men compared to EA men (78.20% vs. 73.28%, *P* < 0.01) (Additional File 1, Table S4; Additional File 2, Figs. S5C–D).

20 samples from AA prostate cancer patients that are present on the TMAs were also analyzed by RNA sequencing. We used these overlapping samples to determine whether AR transcriptional activity is altered. We utilized Gene Set Variation Analysis (GSVA) [42] to derive expression scores for the canonical AR gene targets *KLK2*, *KLK3*, *NKX3.1*, and *TMPRSS2* (measured by RNA sequencing) [55, 56]. GSVA scores can be used to determine pathway activity, and in this case AR transcriptional activity, which provides an advantage over single gene measurements. These GSVA scores indicated AR transcriptional activity is significantly higher in prostate tumors than in adjacent non-tumor tissues (*P* < 0.05) (Additional File 2, Fig. S5E). To add rigor to our study, we expanded our AR activity analysis to the TCGA prostate cancer dataset (EA: *n* = 270 T, 36 NT; AA: *n* = 43 T, 6 NT). The results recapitulate findings from the Roswell Park cohort, where we observed an increase in AR transcriptional activity in tumor versus non-tumor in EA but not AA men (Additional File 2, Fig. S5F). To further investigate race-specific differences in AR activity, we performed GSVA for a 27-gene AR activity score developed by Hieronymus, H. et al. [57] in both the Roswell Park and the TCGA cohorts. AR activity was significantly higher in tumor tissues compared to normal tissues from EA men (Additional File 2, Figs. S5G–H). These results show that AR transcriptional activity is significantly higher in tumors from EA men despite AR protein expression being similar in prostate cancer and adjacent non-tumor tissues. Future studies analyzing AR protein expression and AR transcriptional activity from tumor tissues can provide insights into these observations in EA men. These observations suggest that determining AR protein and target gene expression may reflect AR activity more accurately.

**Fig. 3:**
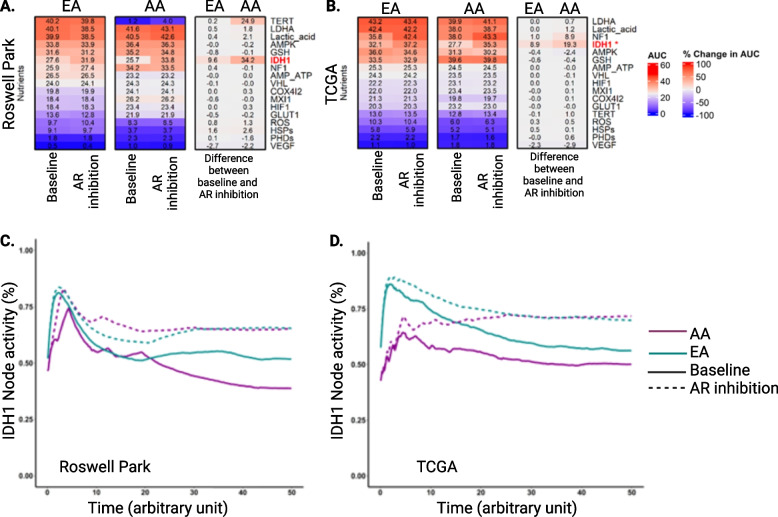
*IDH1* gene activities after AR knockout simulations show significant race-specific differences in prostate tumors. AUC before inhibition (WT), AUC after AR inhibition (KO), and the percent change in AUC between the two conditions in the Nutrient pathway are represented in the heatmap. **A** Roswell Park (RP) clinical samples, **B** TCGA clinical samples. **C**–**D** Percent change in IDH1 AUC (highlighted in red) was significantly different between prostate tumors from AA and EA men in both **C** RP and **D** TCGA clinical samples. Changes in AUC are represented by blue (down) and red (up)

Targeting AR to alter transcriptional signaling and block disease progression is a common therapeutic strategy in prostate cancer [58]. It is unknown whether AR targeting in the clinic results in downstream biological consequences that are different for AA and EA prostate cancer patients. To address this question, we adapted an existing prostate cancer Boolean network [15] to analyze a subset of clinical samples from Roswell Park (*N* = 15, AA: 7, EA: 8; a subset of samples listed in Additional File 1, Table S1). These 15 samples had the three data types (transcriptomics, mutation, and copy number variation) required to generate patient-specific Boolean networks. Men with prostate cancer receive AR-targeted therapies for prolonged periods, usually until the disease relapses [59]. Therefore, we perturbed all patient-specific networks to mimic prolonged AR-targeted therapy for each patient and compared the percent change in AUC of all nodes before and after perturbation between races, in which AUC represents the temporal activity of a node over an arbitrary unit of time. Simulated AR inhibition resulted in significant differences in the changes in AUC of nodes involved in the TGF-β, IDH1, AR, and cell cycle pathways in prostate tumors between AA and EA men (Additional File 2, Figs. S6A–B). A significantly greater increase in AUC profiles was observed for *IDH1* (34.2 vs. 9.6, *P* < 0.05, Fig. 3A, C) and *SMAD* (48.5 vs. 15.3, *P* < 0.05, Additional File 2, Fig. S6A, C, left panels) after AR inhibition in prostate tumors from AA men compared to EA men. Conversely, a significantly greater decrease in AUC profiles was observed for *AR_ERG* (− 97.4 vs. − 93.4, *P* < 0.05) and *ZBTB17* (− 95.8 vs. − 78.5, *P* < 0.05) in AA men (Additional File 1, Table S5). The changes in the AUC of all nodes in this prostate cancer network are represented in Additional File 2, Fig. S6A. These results highlight that there are racial differences in the changes in certain gene activities after prolonged AR inhibition. We validated our findings by simulating AR inhibition with The Cancer Genome Atlas (TCGA) [60] cohort. These simulations confirmed race-specific differences in TGF-β, IDH1, and cell cycle pathways (*P* < 0.05, Fig. 3B, D; Additional File 2, Fig. S6B, D right panels; Additional File 1, Table S5). Overall, our simulations suggest that prolonged AR inhibition can result in changes in gene activity that is significantly different between prostate tumors from AA men and EA men. These observations have real-world implications and need to be investigated in the future since AR inhibition is routinely used to treat advanced prostate cancer.

### Metabolic, oncogenic, and immune pathways are dysregulated in prostate tumors from AA men

**Fig. 4:**
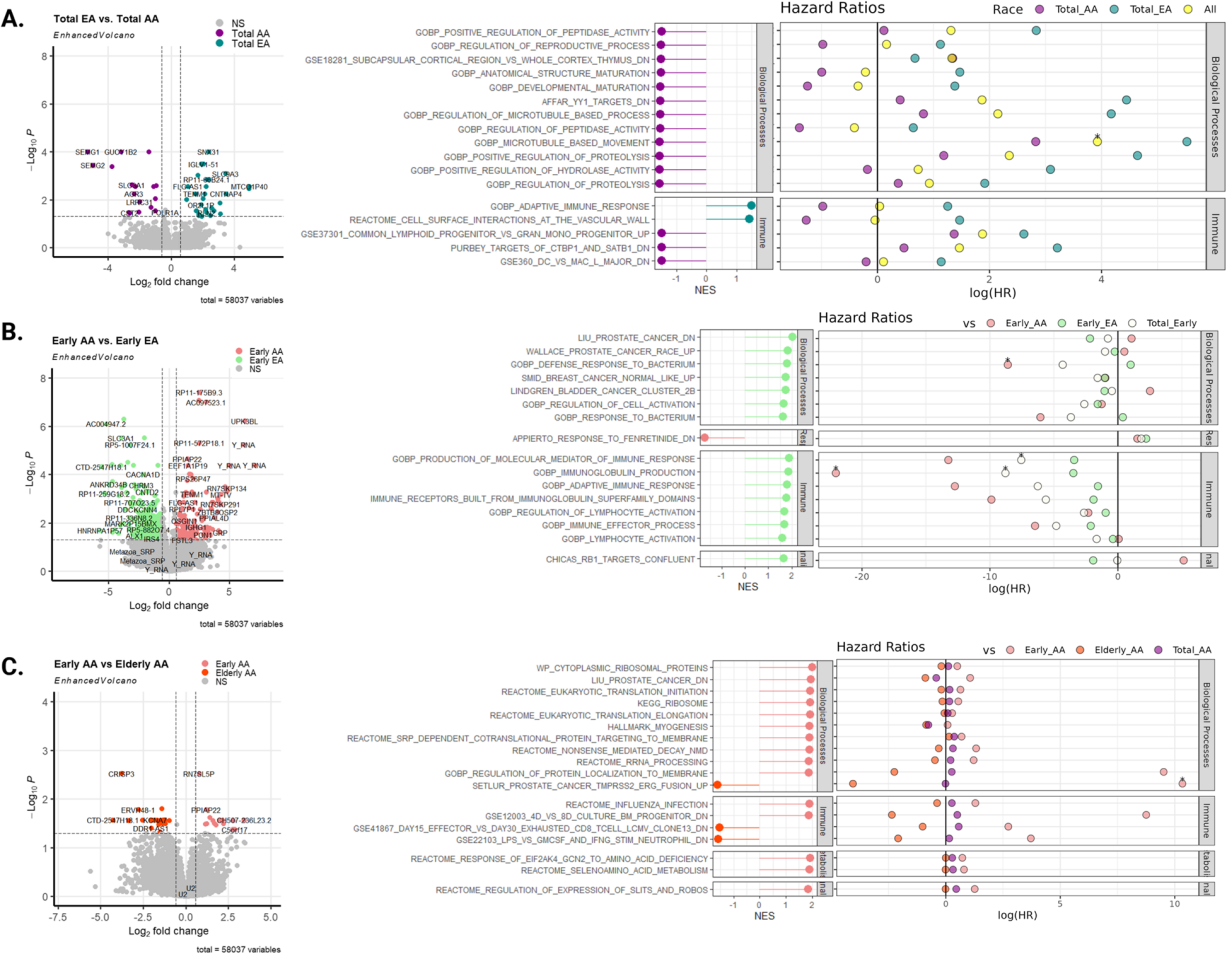
Race comparisons reveal distinct gene enrichments that correlate with progression-free survival. *Left* panels represent differentially expressed genes utilizing DESeq2, *middle* panels represent gene set enrichment analysis based on clusterProfiler, and *right* panels represent hazard ratios based on calculated GSVA scores. **A** Comparison of all prostate tumors from AA and EA men. **B** Race-specific comparisons show distinct pathways in prostate tumors of AA and EA men younger than 55. **C** Comparisons show distinct pathways in prostate tumors of AA men younger than 55 compared to AA men ≥ 55. NES: Normalized Enrichment Score. An asterisk indicates a significant (*p* < 0.05) enrichment of the indicated pathway

Our prostate cancer Boolean network analyses are limited to a subset of curated genes [15]. Broadening this analysis to encompass all gene expression changes allowed us to pinpoint other race-specific differences in prostate cancer transcriptomes. For this purpose, we compared mRNA levels, obtained from RNA sequencing, between prostate tumors of AA and EA men (AA: T = 31 and EA: T = 32, demographics in Additional File 1, Table S2, samples that were also analyzed by DNA methylation). Genes in peptidase activity pathways, including *SEMG1* and *SEMG2,* were significantly upregulated in prostate cancer from AA men (Fig. 4A, left panel, Additional File 5). To identify race-specific pathway alterations, we performed GSEA using the whole transcriptome data. GSEA revealed peptidase activity, reproductive processes, and microtubule-based movement were overrepresented in prostate tumors from AA men (Fig. 4A, middle panel). Additionally, immune-based pathways that include genes crucial for the proliferation of lymphoid and T-cell progenitors, such as *CD40* and *CD226*, were enriched in prostate tumors from AA men (Fig. 4A, middle panel). The results from the Roswell Park cohort are further supported by the analysis we conducted on the larger TCGA cohort (*n* = 43 AA; *n* = 270 EA), which confirmed enrichment of these pathways in AA prostate tumors (Additional File 2, Fig. S7A).

Gene expression panels based on measuring expression of multiple genes on one panel, such as Decipher [61], can be utilized to derive an overall score for calculating the risk of prostate cancer progression. Therefore, we looked at gene expression in each individual pathway as a potential candidate for ‘gene marker panels’ and calculated GSVA scores. GSVA estimates variation of a gene set and calculates sample-wise (in this case for tumors from individual patients) scores as a function of genes within the given GSEA pathway [42]. While GSEA reveals the enriched pathway, GSVA can be thought of as “activity score” for the genes included in the enriched pathways. Therefore, we calculated GSVA scores for the top pathways identified using GSEA. Using the GSVA scores we determined hazard ratios (HR) based on progression-free survival. Positive HR scores indicate a higher risk of disease progression, and negative HR scores indicate a reduced risk of disease progression. Higher GSVA scores in the microtubule-based movement pathway correlated with positive HR scores and therefore higher risk of disease progression in both AA and EA men (*P* < 0.05) (Fig. 4A, right panel; Additional File 2, Fig. S7B; Additional File 1, Table S6). The microtubule-based movement pathway included multiple members of the kinesin family that are known to promote disease progression in other cancers [62]. These results lay the groundwork to further develop and test gene expression of the microtubule-based movement pathway in primary prostate cancer and its association with disease progression.

AA men are more frequently diagnosed with early-onset prostate cancer (< 55 years of age) than EA men [63]. To determine race-specific gene expression changes associated with early-onset disease, we identified differentially expressed genes (DEGs) in prostate cancer from < 55 years and ≥ 55 years AA and EA men (Additional File 2, Fig. S7C). Components of the ribonucleoprotein (*RN7SK* genes) and *Y RNA*, part of long noncoding RNAs, were significantly upregulated in prostate cancer from AA men < 55 years compared to EA men < 55 years (Fig. 4B, left panel). GSEA revealed an overrepresentation of genes that include enzymes involved in amino-acid metabolism, oxidation of organic compounds, and oxidative phosphorylation (Appierto response in Fig. 4B, middle panel). We again calculated HRs based on progression-free survival and associated GSVA scores to determine whether these pathways correlate with the risk of disease progression. Downregulation of the defense response to bacterium and immunoglobulin production pathways was associated with a significantly lower risk of disease progression only in younger AA men (*P* < 0.05, Fig. 4B, right panel; Additional File 2, Fig. S7B). Overtreatment of men with low-risk primary prostate cancer remains a significant issue in the clinic [64]. Therefore, our finding represents a potential path forward for identifying AA men with primary prostate cancer with lower risk of disease progression by measuring gene expression changes in the bacterium and immunoglobulin pathways.

To determine tumor-specific gene expression changes, we identified DEGs in prostate cancer from men < 55 years of age and men ≥ 55 years of age by comparing tumors and adjacent non-tumor tissue within each race. *RN7SL5P*, a long noncoding RNA, as well as other genes, was significantly upregulated in prostate cancer from AA men < 55 years compared to AA men ≥ 55 years (Fig. 4C, left panel; Additional File 5). GSEA revealed that early-onset prostate cancer in AA men was enriched for ribosomal proteins, translational, and metabolism-associated pathways (Fig. 4C, middle panel; Additional File 1, Table S6). Lower GSVA scores of the TMPRSS2-FUSION pathway were associated with a higher risk of disease progression only in AA men < 55 years of age but not in AA men ≥ 55 years of age (Fig. 4C, right panel; Additional File 2, Fig. S7B; Additional File 1, Table S6). Interestingly, we did not identify any significant DEGs or pathways associated with disease recurrence upon comparisons of EA men < 55 years of age and EA men ≥ 55 years of age (Additional File 2, Fig. S7D), suggesting that individual gene expression is similar between these two cohorts. Overall, we identify distinct transcriptional changes associated with the risk of disease progression in AA and EA men with prostate cancer. Future studies of these signaling pathways in preclinical models should pinpoint their mechanistic contributions to prostate cancer biology and disease progression.

## Discussion

The significant underrepresentation of clinical samples from AA men [60, 65], including larger datasets like the TCGA, has contributed to the limited understanding of the mechanisms that influence disparities in prostate cancer biology. This exclusion is particularly evident for prostate cancer in AA men < 55 years old, who have an increased frequency of prostate cancer compared to EA men < 55 years old [63]. We start to address this significant gap by studying prostate tissues from AA men with prostate cancer. Our study utilized tumors from self-identified patients treated at Roswell Park. We previously performed ancestry informative marker analyses for a subset of samples included in these analyses, which showed concordance between self-identification and ancestry [66].

Our results highlight gene expression changes that correlate with progression-free survival in AA men < 55 years old. We characterized paired clinical samples of prostate cancer and adjacent non-tumor tissues through DNA methylation arrays, RNA sequencing, and immunohistochemistry studies. Performing these analyses on paired tissues from the same individual allowed us to compare alterations to adjacent non-tumor tissues and dissect tumor-specific molecular changes. Furthermore, by analyzing adjacent non-tumor tissues, we were able to characterize molecular changes that exist in histologically normal prostate tissues.

Our Principal Component Analysis (PCA) plots and direct comparisons of DMRs show that DNA hypermethylation is similar between prostate cancer and adjacent non-tumor tissues from AA men. We discovered multiple DNA methylation changes in adjacent non-tumor tissues as well as prostate cancer tissues from AA men. These DNA methylation alterations correlate with gene expression in a race-specific manner. For example, DNA methylation at transcription start sites and 5′-UTR regions is distinctly associated with gene expression of the GATA family of transcription factors in adjacent non-tumor tissues of AA men. These changes in DNA methylation and gene expression happen before any obvious change in histology. This is a clinically significant finding for prostate cancer diagnoses, which currently relies on histological examination of tissue biopsies. Biopsies that miss the transformed tissue and sample histologically normal-looking tissue can result in false negative diagnosis (~25%), especially in the context of rising PSA measurements [67]. False negatives can hinder timely diagnosis and follow-up care, which can have particularly harmful effects on younger AA men, who are diagnosed at a younger age and present with high-grade tumors [63]. Our observations of DNA methylation changes at specific loci in histologically normal-looking adjacent non-tumor tissues suggest that quantifying DNA methylation can be developed for diagnostic and prognostic purposes in prostate cancer. A previous study measuring DNA methylation at *GSTP1*, *APC*, and *RASSF1* loci in repeat prostate biopsies identified AA and EA men at risk of high-grade prostate cancer [68]. This test was performed with > 60% sensitivity and 70% specificity for men younger than 55 years [68]. Altogether, we provide a foundation and further rationale for developing and testing DNA methylation measurements at specific loci, including those in *GATA* gene regions. Our observations also suggest a role for DNA methylation regulating gene expression of GATA family members that are known AR co-regulators [51], and therefore potentially involved in underlying disease biology.

DNA methylation can modify the composition of transcription factors and chromatin remodeling enzymes at specific loci to regulate gene transcription and, therefore, expression [69]. Cofactors that regulate gene transcription with DNA methylation have been previously identified in prostate cancer [14]. Whether these associations occur in a race-specific manner was previously unknown. The results of this study indicate that DNA methylation cooperates with distinct transcriptional coregulators in prostate cancer from AA men compared to EA men. We show that hypomethylated regions are enriched in transcriptional regulators, including CTCF, only in prostate tumors of AA men. CTCF loss leads to hypermethylation at its binding sites and is associated with decreased gene expression in prostate epithelial cells [70]. This, along with our findings, suggests the enrichment of CTCF in DNA hypomethylated regions can potentially regulate gene expression specifically in prostate tumors of AA men and will be investigated in the future.

We find that gene expression of several androgen receptor (AR) target genes, including *TRIM63*, are inversely associated with DNA methylation only in prostate cancer from AA men. Therefore, we used a unique systems pharmacology approach to investigate the broad effects of AR-mediated gene expression changes on prostate cancer from AA and EA men. The patient-specific prostate cancer Boolean networks allowed us to closely mimic clinical disease with multiple genetic alterations and copy number alterations. We simulated transcriptional alterations that arise from prolonged AR inhibition in prostate cancer and compared these alterations between AA and EA men. Our simulation using primary therapy naïve tumors suggests that prolonged AR inhibition in AA men is more likely to result in therapy-resistant phenotypes associated with TGF-β (SMAD), IDH1, and cell cycle pathways [71–73]. These observations are clinically significant for AA men, who are more frequently diagnosed with prostate cancer at a younger age (< 55 years) than EA men [6, 63], and are more likely to remain on a life-long AR-targeted therapy. Our observations provide a rationale to measure gene expression changes in TGF-β, IDH1, and cell cycle pathways, in prostate cancer of AA and EA men treated with AR inhibitors. Measuring gene expression changes upon AR-targeted therapy in AA and EA men can provide insights into race-specific mechanisms and perhaps markers of therapy response and emerging resistance.

Prostate cancer from EA men often contains ERG fusion/ETS family fusions and SPINK1 mutations [74, 75], which are not as commonly found in prostate cancer from AA men. Measuring gene expression changes in prostate cancer from AA men represents an important tool for the clinical management of prostate cancer as they typically lack the currently known genetic aberrations. Genomic classification that is based on gene expression measured in radical prostatectomy specimens, including Decipher, is currently utilized as an additional tool for prognostic purposes [61]. Our study highlights gene expression changes occurring in AA men as well as AA men < 55 years of age. We find that upregulation of TMPRSS2_ERG-associated gene signatures was associated with a shorter time to progression-free survival in AA men < 55 years of age compared to AA men ≥ 55 years of age. We also show that downregulation of immune-related signaling is associated with longer progression-free survival in AA men < 55 years of age compared to EA men < 55 years of men. Therefore, the current study can lead to the development of newer assays in addition to existing tools that are specifically designed to improve the clinical management of prostate cancer in AA men.

## Conclusions

In conclusion, our study reveals significant differences in the molecular landscape of prostate cancer between African American (AA) and European American (EA) men, especially in those under 55 years of age. We observe distinct DNA methylation patterns at specific loci, suggesting unique epigenetic regulatory mechanisms at play in prostate tumors from AA men tumors compared to EA men. Moreover, the composition of gene transcription coregulators differs between the two racial groups, indicating potential divergent pathways of disease progression. Importantly, our study identifies race-specific gene expression profiles, particularly in genes associated with androgen receptor signaling, immune response, and pathways implicated in therapy resistance. Through patient-specific prostate cancer Boolean network modeling, we uncover race-specific transcriptional alterations, offering insights into potential mechanisms of therapy resistance in AA men. These findings underscore the importance of considering racial disparities in prostate cancer biology and highlight the need for personalized approaches in diagnosis, prognosis, and treatment. Further validation and exploration of these molecular differences hold promise for improving clinical management and outcomes, particularly for AA men, in the context of prostate cancer.

### Supplementary Information

**Additional File 1.**

**Additional File 2.**

**Additional File 3.**

**Additional File 4.**

**Additional File 5.**

**Additional File 6.**

## Data Availability

The sequencing data generated in this publication have been deposited in NCBI's Gene Expression Omnibus and are accessible through GEO Series accession numbers GSE237995 (https://www.ncbi.nlm.nih.gov/geo/query/acc.cgi?acc=GSE237995), GSE262522 (https://www.ncbi.nlm.nih.gov/geo/query/acc.cgi?acc=GSE262522), and  GSE262524 (https://0-www-ncbi-nlm-nih-gov.brum.beds.ac.uk/geo/query/acc.cgi?acc=GSE262524) [43].
