# Supplementary material for: Race-specific coregulatory and transcriptomic profiles associated with DNA methylation and androgen receptor in prostate cancer
Source: Genome Med. 2024 Apr 2;16:52. doi: 10.1186/s13073-024-01323-6 (PMC10988846; doi:10.1186/s13073-024-01323-6)
Supplement: Supplementary file 1 — Additional File 1. [file 13073_2024_1323_MOESM1_ESM.pdf]

Table S1 Demographics for all samples analyzed for DNA methylation and RNA sequencing

		**AA N(%)**	**EA N(%)**	**Overall**	***P*-value**
Overall	N (%)	35 (46.1)	41 (53.9)	76 (100%)	
Age at Diagnosis	Mean/Std/N	57.2/7.9/35	57.8/6.6/41	57.5/7.2/76	0.707
	Median/Min/Max	57.0/44.0/73.0	57.0/45.0/72.0	57.0/44.0/73.0	
Smoking Status	Never	13 (37.1%)	18 (43.9%)	31 (40.8%)	0.133
	Former	11 (31.4%)	18 (43.9%)	29 (38.2%)	
	Current	11 (31.4%)	5 (12.2%)	16 (21.1%)	
Cliical Gleason score	< 7	8 (22.9%)	12 (29.3%)	20 (26.3%)	0.724
	7	21 (60.0%)	24 (58.5%)	45 (59.2%)	
	> 7	6 (17.1%)	5 (12.2%)	11 (14.5%)	
Pre-RP PSA	Mean/Std/N	11.0/14.1/35	8.9/6.1/41	9.8/10.6/76	0.621
	Median/Min/Max	6.9/2.8/82.1	6.4/1.7/25.9	6.9/1.7/82.1	
PSA*	< 10	29 (82.9%)	29 (70.7%)	58 (76.3%)	0.221
	10 to <20	2 (5.7%)	8 (19.5%)	10 (13.2%)	
	>= 20	4 (11.4%)	4 (9.8%)	8 (10.5%)	
Clinical T-stage	cT1	19 (54.3%)	23 (56.1%)	42 (55.3%)	1.000
	cT2	14 (40.0%)	16 (39.0%)	30 (39.5%)	
	cT3	2 (5.7%)	2 (4.9%)	4 (5.3%)	
Grade	II	4 (11.4%)	6 (15.0%)	10 (13.3%)	0.742
	III	31 (88.6%)	34 (85.0%)	65 (86.7%)	
Pathological Gleason	< 7	4 (11.4%)	4 (9.8%)	8 (10.5%)	0.814
	7	28 (80.0%)	35 (85.4%)	63 (82.9%)	
	> 7	3 (8.6%)	2 (4.9%)	5 (6.6%)	
Pathological T-stage	pT2	19 (54.3%)	22 (53.7%)	41 (53.9%)	0.271
	pT3	12 (34.3%)	18 (43.9%)	30 (39.5%)	
	pT4	4 (11.4%)	1 (2.4%)	5 (6.6%)	

Table S2 Demographics for overlapping samples analyzed by both DNA methylation and RNA sequencing

		EA N(%)	AA N(%)	Overall	*P* -value
Overall	N	32 (50.8)	31 (49.2)	63 (100%)	
Age at Diagnosis	Mean/Std/N	58.3/6.7/32	56.4/7.5/31	57.4/7.1/63	0.321
	Median/Min/Max	57.5/45.0/72.0	56.0/44.0/73.0	57.0/44.0/73.0	
Smoking Status	Never	15 (46.9%)	12 (38.7%)	27 (42.9%)	0.039
	Former	14 (43.8%)	8 (25.8%)	22 (34.9%)	
	Current	3 (9.4%)	11 (35.5%)	14 (22.2%)	
Cliical Gleason score	< 7	9 (28.1%)	8 (25.8%)	17 (27.0%)	0.819
	7	20 (62.5%)	18 (58.1%)	38 (60.3%)	
	> 7	3 (9.4%)	5 (16.1%)	8 (12.7%)	
Pre-RP PSA	Mean/Std/N	8.6/5.7/32	10.8/14.7/31	9.7/11.1/63	0.815
	Median/Min/Max	6.7/1.7/25.9	6.9/2.8/82.1	6.9/1.7/82.1	
PSA*	< 10	22 (68.8%)	26 (83.9%)	48 (76.2%)	0.141
	20-Oct	8 (25.0%)	2 (6.5%)	10 (15.9%)	
	>= 20	2 (6.3%)	3 (9.7%)	5 (7.9%)	
Clinical T-stage	cT1	19 (59.4%)	16 (51.6%)	35 (55.6%)	0.845
	cT2	11 (34.4%)	13 (41.9%)	24 (38.1%)	
	cT3	2 (6.3%)	2 (6.5%)	4 (6.3%)	
Grade	II	5 (16.1%)	4 (12.9%)	9 (14.5%)	1
	III	26 (83.9%)	27 (87.1%)	53 (85.5%)	
Pathological Gleason	< 7	4 (12.5%)	4 (12.9%)	8 (12.7%)	0.903
	7	26 (81.3%)	24 (77.4%)	50 (79.4%)	
	> 7	2 (6.3%)	3 (9.7%)	5 (7.9%)	
Pathological T-stage	pT2	17 (53.1%)	18 (58.1%)	35 (55.6%)	0.232
	pT3	14 (43.8%)	9 (29.0%)	23 (36.5%)	
	pT4	1 (3.1%)	4 (12.9%)	5 (7.9%)	

Table S3. Demographics in TMA

		AA N (%)	EA N (%)	Overall	*P* -value
Overall	N	107 (44.6)	133 (55.4)	253 (100%)	
Age at Diagnosis	Mean/Std/N	56.0/7.1/107	59.7/6.7/133	58.1/7.1/240	<.001
	Median/Min/Max	55.0/41.0/70.0	60.0/41.0/78.0	58.0/41.0/78.0	
Age*	< 55	46 (43.0%)	29 (21.8%)	80 (31.6%)	<.001
	>= 55	61 (57.0%)	104 (78.2%)	173 (68.4%)	
Tobacco Use	None	42 (39.3%)	49 (37.4%)	92 (38.5%)	<0.01
	Former	32 (29.9%)	63 (48.1%)	95 (39.7%)	
	Current	33 (30.8%)	19 (14.5%)	52 (21.8%)	
pre-RP PSA	Mean/Std/N	9.1/10.3/106	9.1/10.7/132	9.1/10.5/238	0.48
	Median/Min/Max	6.5/0.7/82.1	6.3/0.1/87.9	6.3/0.1/87.9	
PSA*	< 4	16 (15.1%)	24 (18.2%)	40 (16.7%)	0.92
	4 to <10	70 (66.0%)	82 (62.1%)	152 (63.6%)	
	10 to <20	12 (11.3%)	16 (12.1%)	28 (11.7%)	
	>= 20	8 (7.5%)	10 (7.6%)	19 (7.9%)	
Grade	Grade II	32 (29.9%)	33 (24.8%)	65 (25.7%)	0.39
	Grade III	75 (70.1%)	100 (75.2%)	188 (74.3%)	
Clinical Gleason	<= 3+4	61 (57.0%)	76 (58.0%)	138 (57.7%)	0.90
	>= 4+3	46 (43.0%)	55 (42.0%)	101 (42.3%)	
Clinical T-stage	cT1	71 (68.9%)	90 (68.7%)	162 (68.9%)	0.53
	cT2	28 (27.2%)	39 (29.8%)	67 (28.5%)	
	cT3	4 (3.9%)	2 (1.5%)	6 (2.6%)	
Path Gleason	< 3+4	45 (42.5%)	49 (37.1%)	94 (39.3%)	0.43
	> 4+3	61 (57.5%)	83 (62.9%)	145 (60.7%)	
Path T-stage	pT2	71 (68.9%)	75 (57.7%)	146 (62.4%)	0.23
	pT3	27 (26.2%)	46 (35.4%)	74 (31.6%)	
	pT4	5 (4.9%)	9 (6.9%)	14 (6.0%)	
Persistent Disease?	No	84 (78.5%)	106 (79.7%)	200 (79.1%)	0.87
	Yes	23 (21.5%)	27 (20.3%)	53 (20.9%)	

Table S4. Percent AR positive nuclei in prostate tissues from AA and EA men

Percent of AR+ve nuclei		AA			EA			Interaction *P*-value
**Tumor**		**n**	**Mean (SD)**	***P*-value**	**n**	**Mean (SD)**	***P*-value**	
Age (range)	<55	46	77.59 (11.80)	0.49	29	80.63 (13.93)	0.07	0.28
	≥ 55	61	75.80 (14.28)		104	74.07 (17.86)		
PSA (range, ng/mL)	<4	16	72.18 (13.65)	0.15	24	75.47 (13.15)	0.96	0.38
	4 to <10	70	77.91 (12.02)		82	75.18 (18.91)		
	10 to <20	12	72.53 (19.51)		16	77.68 (15.49)		
	≥ 20	8	82.51 (5.55)		10	74.49 (17.08)		
Clinical Gleason score	≤ 3+4	61	77.61 (12.30)	0.35	76	73.10 (17.07)	0.06	<0.05
	≥ 4+3	46	75.19 (14.43)		55	78.82 (17.40)		
Pathological T-stage	pT2	71	77.21 (12.59)	0.87	75	75.44 (15.87)	0.7	0.70
	pT3	27	78.58 (11.72)		46	74.41 (19.98)		
	pT4	5	76.52 (9.36)		9	79.72 (9.47)		
Persistent disease	No	84	76.34 (12.96)	0.74	106	75.20 (17.17)	0.69	0.93
	Yes	23	77.39 (14.49)		27	76.70 (17.81)		
Hormone therapy after radical prostatectomy	No	86	76.62 (13.26)	0.93	100	75.32 (17.07)	0.83	0.84
	Yes	21	76.35 (13.49)		33	76.06 (18.02)		

Percent of AR+ve nuclei		AA			EA			Interaction *P*-value
**Adjacent non-tumor**		**n**	**Mean (SD)**	***P*-value**	**n**	**Mean (SD)**	***P*-value**	
Age (range)	<55	46	80.19 (9.90)	0.16	29	78.09 (16.58)	<0.05	0.22
	≥ 55	61	76.63 (14.82)		104	68.68 (19.28)		
PSA (range, ng/mL)	<4	16	74.41 (14.14)	<0.05	24	69.00 (12.61)	0.97	0.5
	4 to <10	70	80.57 (10.70)		82	71.06 (20.26)		
	10 to <20	12	70.36 (21.56)		16	70.71 (20.98)		
	≥ 20	8	75.48 (7.94)		10	71.38 (21.87)		
Clinical Gleason score	≤ 3+4	61	76.66 (12.55)	0.17	76	69.95 (19.27)	0.69	0.63
	≥ 4+3	46	80.15 (13.45)		55	71.31 (19.07)		
Pathological T-stage	pT2	71	79.78 (11.40)	0.28	75	71.28 (13.34)	0.84	0.53
	pT3	27	79.82 (9.59)		46	69.34 (25.53)		
	pT4	5	71.62 (14.02)		9	72.18 (24.53)		
Persistent disease	No	84	79.27 (11.86)	0.09	106	70.05 (18.91)	0.42	0.11
	Yes	23	74.09 (16.18)		27	73.39 (19.80)		
Hormone therapy after radical prostatectomy	No	86	80.45 (10.74)	<0.001	100	70.59 (17.92)	0.88	<0.05
	Yes	21	68.78 (17.03)		33	71.15 (22.47)		

PSA: prostate specific antigen, n: number of samples, SD: standard deviation

Table S5. Differentially expressed nodes after AR KO between AA and EA men

Node (Roswell Park cohort)	Pathway	% Change in AUC in AA men	%Change in AUC in EA men	*P* -value	Adjusted *P* -value
		Median (Q1 – Q3)	Median (Q1 – Q3)		
SMAD	TGFb pathway	48.5 (43.3 – 120.3)	15.3 (3.6 – 46.3)	0.0401	0.12
IDH1	Nutrient pathway	34.2 (17.8 – 47.7)	9.6 (3.94 – 13.5)	0.00373	0.0629
AR_ERG	AR pathway	-97.4 (-98.4 – -96.2)	-93.4 (-95.6 – -82.7)	0.0140	0.041
ZBTB17	Cell cycle	-95.8 (-97.8 – -89.0)	-78.5 (-84.9 – -50.6)	0.0401	0.252

Node (TCGA)	Pathway	% Change in AUC in AA men	%Change in AUC in EA men	*P* -value	Adjusted *P* -value
		Median (Q1 – Q3)	Median (Q1 – Q3)		
SMAD	TGFb pathway	51.9 (33.3 – 76.4)	28.0 (12.1 – 61.3)	0.0108	0.0024
IDH1	Nutrient pathway	19.9 (8.83 – 36.3)	8.90 (1.88 – 20.7)	0.000313	0.0051
E2F1	Cell cycle	-96.8 (-98.4 – -88.0)	-91.0 (-97.7 – -67.1)	0.000817	0.0378
Beta catenin	WNT pathway	-89.5(-95.4 – -44.2)	-65.7 (-88.3 – -24.8)	0.0153	0.107

Table S6. Top GSEA pathways with hazard ratios based on GSVA scores for early/elderly and race comparisons

Comparisons	Names	NES	Early_AA.HR	Early_AA.SE	Early_AA.P-value	Early_EA.HR	Early_EA.SE	Early_EA.P-value	Total_Early.HR	Total_Early.SE	Total_Early.P-value
EarlyAA_vs_EA	APPIERTO_RESPONSE_TO_FENRETINIDE_DN	-1.73628536	4.570173212	2.10066837	0.461594168	9.076245096	2.985163364	0.451827676	6.203982357	1.561071561	0.234006439
EarlyAA_vs_EA	GOBP_LYMPHOCYTE_ACTIVATION	1.58366032	1.060683624	5.052465866	0.990696563	0.677593657	4.122599071	0.924753014	0.189564927	2.715138611	0.537775099
EarlyAA_vs_EA	GOBP_RESPONSE_TO_BACTERIUM	1.63698156	0.002353902	3.972493024	0.101219669	1.467932804	3.903465423	0.921630689	0.02539631	2.583783726	0.143878847
EarlyAA_vs_EA	GOBP_REGULATION_OF_CELL_ACTIVATION	1.66919426	0.281414147	5.121817266	0.804468055	0.205775966	4.21650638	0.705769619	0.072415062	2.819723235	0.345994992
EarlyAA_vs_EA	CHICAS_RB1_TARGETS_CONFLUENT	1.67065976	169.4138354	4.170452638	0.215748342	0.145078565	3.562698961	0.579248004	0.953201534	1.880818864	0.979669486
EarlyAA_vs_EA	GOBP_IMMUNE_EFFECTOR_PROCESS	1.67577576	0.001546753	5.273659709	0.203025801	0.11968747	4.976739564	0.667197379	0.008094832	3.212057523	0.121287609
EarlyAA_vs_EA	GOBP_REGULATION_OF_LYMPHOCYTE_ACTIVATION	1.6770448	0.099293477	5.104007542	0.650857328	0.384039424	4.128325408	0.816207269	0.070744349	2.867431469	0.349996731
EarlyAA_vs_EA	LINDGREN_BLADDER_CANCER_CLUSTER_2B	1.74925605	12.25311649	3.197528808	0.433779491	0.35133334	2.715124722	0.697929561	0.62531909	1.579665727	0.766026923
EarlyAA_vs_EA	SMID_BREAST_CANCER_NORMAL_LIKE_UP	1.75778191	0.373926305	2.859903397	0.730403854	0.359282954	2.688547551	0.701584487	0.204249457	1.572775818	0.305026066
EarlyAA_vs_EA	GOBP_ADAPTIVE_IMMUNE_RESPONSE_BASED_ON_SOMATIC_RECOMBINATION_OF_IMMUNE_RECEPTORS_BUILT_FROM_IMMUNOGLOBULIN_SUPERFAMII	1.76452569	5.09693E-05	6.521208638	0.114533204	0.148874303	5.115798655	0.708199245	0.003603108	3.579174107	0.105745375
EarlyAA_vs_EA	GOBP_ADAPTIVE_IMMUNE_RESPONSE	1.78940266	2.92633E-06	7.396683205	0.054490742	0.21009597	4.502212896	0.727899447	0.001913456	3.491438512	0.063544824
EarlyAA_vs_EA	GOBP_DEFENSE_RESPONSE_TO_BACTERIUM	1.79841331	0.000184137	5.125988426	0.033135749	2.698540139	3.688369055	0.787198179	0.013420114	2.574267083	0.073424544
EarlyAA_vs_EA	WALLACE_PROSTATE_CANCER_RACE_UP	1.84246311	1.650248589	3.632765842	0.890284664	0.780975228	2.881103361	0.931607741	0.366076357	1.730376323	0.55954006
EarlyAA_vs_EA	GOBP_IMMUNOGLOBULIN_PRODUCTION	1.87102769	2.6844E-10	14.23564561	0.048742205	0.030753602	4.893670944	0.470613277	0.000152452	4.51009376	0.038683384
EarlyAA_vs_EA	GOBP_PRODUCTION_OF_MOLECULAR_MEDIATOR_OF_IMMUNE_RESPONSE	1.87539726	1.75585E-06	7.945787636	0.069751759	0.032789718	4.977904336	0.48530327	0.000525888	3.971593058	0.047622627
EarlyAA_vs_EA	LIU_PROSTATE_CANCER_DN	2.03563015	2.894919761	1.883144727	0.565662622	0.112798912	2.279594985	0.269772645	0.457312201	1.021828612	0.437544982
Comparisons	Names	NES	Elderly_AA.HR	Elderly_AA.SE	Elderly_AA.P-valu	Early_AA.HR	Early_AA.SE	Early_AA.P-value	Total_AA.HR	Total_AA.SE	Total_AA.P-value
Early_vs_ElderlyAA	SETLUR_PROSTATE_CANCER_TMPRSS2_ERG_FUSION_UP	-1.60703165	0.984877272	1.218642874	0.990023274	13.81250944	1.325159688	0.02939409	2.809107395	0.810079802	0.197099274
Early_vs_ElderlyAA	GSE22103_LPS_VS_GMCSF_AND_IFNG_STIM_NEUTROPHIL_DN	-1.58762297	1.159809014	1.338442017	0.9117866	78.07475983	2.977611088	0.127753104	2.919317666	1.245421938	0.38770012
Early_vs_ElderlyAA	GSE41867_DAY15_EFFECTOR_VS_DAY30_EXHAUSTED_CD8_TCELL_LCMV_CLONE13_DN	-1.53971543	1.754741018	1.159960841	0.626876899	2.857614368	1.546380062	0.492511518	2.494559467	0.923074448	0.318403095
Early_vs_ElderlyAA	REACTOME_REGULATION_OF_EXPRESSION_OF_SLITS_AND_ROBOS	1.86667941	1.574112999	1.108596985	0.681651486	0.713371956	1.473423577	0.818460002	1.322028063	0.873389547	0.749027011
Early_vs_ElderlyAA	GSE12003_4D_VS_8D_CULTURE_BM_PROGENITOR_DN	1.87374033	1.649209943	1.6540272	0.762029673	0.935690516	3.003355371	0.982342516	1.444132603	1.541582001	0.81152343
Early_vs_ElderlyAA	GOBP_REGULATION_OF_PROTEIN_LOCALIZATION_TO_MEMBRANE	1.87546966	1.295362064	1.838143741	0.888003108	8.733642715	2.842734959	0.442268829	2.256505946	1.55120301	0.599118051
Early_vs_ElderlyAA	REACTOME_RRNA_PROCESSING	1.88629906	1.229528462	1.025233816	0.840184593	1.810648341	1.238751895	0.629759219	1.596776839	0.777902698	0.546140279
Early_vs_ElderlyAA	REACTOME_SELENOAMINO_ACID_METABOLISM	1.89405869	1.366224023	0.942151527	0.740075317	1.009523391	1.108953374	0.993180475	1.317703051	0.696585015	0.691626612
Early_vs_ElderlyAA	REACTOME_NONSENSE_MEDIATED_DECAY_NMD	1.89675084	1.363719433	0.913520687	0.733729961	1.127716049	1.115436968	0.914162746	1.362646242	0.696077034	0.656044394
Early_vs_ElderlyAA	REACTOME_INFLUENZA_INFECTION	1.89978014	1.300637038	1.002719696	0.793020493	1.438614363	1.213030264	0.763790734	1.47476459	0.76165468	0.609153959
Early_vs_ElderlyAA	REACTOME_SRP_DEPENDENT_COTRANSLATIONAL_PROTEIN_TARGETING_TO_MEMBRANE	1.89998684	1.441480743	0.876981097	0.675924405	1.309849568	1.029486822	0.792805452	1.495793493	0.649889695	0.534065771
Early_vs_ElderlyAA	HALLMARK_MYOGENESIS	1.90970734	0.466107984	1.318280177	0.561200846	0.330996883	1.948514438	0.56779395	0.467655583	1.091020543	0.484661333
Early_vs_ElderlyAA	REACTOME_RESPONSE_OF_EIF2AK4_GCN2_TO_AMINO_ACID_DEFICIENCY	1.93218744	1.323874505	0.884126327	0.750623021	1.066318488	1.072429378	0.952250364	1.297501233	0.669132947	0.69669421
Early_vs_ElderlyAA	REACTOME_EUKARYOTIC_TRANSLATION_ELONGATION	1.93564597	1.085520034	0.886310661	0.926223615	0.917620825	1.074634902	0.936225838	1.08111773	0.670236759	0.907347129
Early_vs_ElderlyAA	KEGG_RIBOSOME	1.94210712	1.168137057	0.889569433	0.861251332	0.986492326	1.057947936	0.989743571	1.172907962	0.664149268	0.810121083
Early_vs_ElderlyAA	REACTOME_EUKARYOTIC_TRANSLATION_INITIATION	1.94295369	1.178877907	0.877714955	0.851198975	1.302655257	1.037910114	0.798573395	1.306428915	0.658764922	0.684445584
Early_vs_ElderlyAA	LIU_PROSTATE_CANCER_DN	1.95087006	0.65776979	0.917574012	0.646928979	0.140375019	1.596346117	0.18206399	0.458653661	0.719437609	0.2721799
Early_vs_ElderlyAA	WP_CYTOPLASMIC_RIBOSOMAL_PROTEINS	1.9978593	1.11516077	0.90145327	0.903738354	0.943366312	1.094348161	0.957509957	1.116597123	0.682298532	0.871558026
Comparisons	Names	NES	Elderly_EA.HR	Elderly_EA.SE	Elderly_EA.P-valu	Early_EA.HR	Early_EA.SE	Early_EA.P-value	Total_EA.HR	Total_EA.SE	Total_EA.P-value
Early_vs_ElderlyEA	GSE13485_CTRL_VS_DAY7_YF17D_VACCINE_PBMC_UP	-3.154753	0.819607401	3.294960627	0.951847619	0.043051669	4.042445515	0.423156726	1.090808076	2.308900418	0.969969743
Early_vs_ElderlyEA	GSE16450_CTRL_VS_IFNA_12H_STIM_IMMATURE_NEURON_CELL_LINE_DN	-3.15219536	0.276880067	3.634742211	0.722638315	19.24011971	3.852743652	0.431825036	3.33296787	2.478177798	0.625761028
Early_vs_ElderlyEA	GSE29617_CTRL_VS_DAY7_TIV_FLU_VACCINE_PBMC_2008_DN	-3.15130149	16.87769826	3.33546321	0.380623881	5.607018268	2.61712865	0.496772674	10.7869829	1.877501171	0.184248566
Early_vs_ElderlyEA	GSE17721_PAM3CSK4_VS_GADIQUIMOD_6H_BMDC_UP	-3.14858234	1.951308504	3.30491752	0.839450591	2.405808852	2.898021172	0.760803429	4.373149646	1.959388735	0.44590353
Early_vs_ElderlyEA	GSE17721_0.5H_VS_8H_CPG_BMDC_DN	-3.14807633	0.826911353	2.926190271	0.948202838	0.6969303	4.833958546	0.940440997	1.904820948	2.694306682	0.810768408
Early_vs_ElderlyEA	GSE17974_IL4_AND_ANTI_IL12_VS_UNTREATED_48H_ACT_CD4_TCELL_DN	-3.14762996	1.376316213	3.155399374	0.919342382	2.90810092	3.507591674	0.759521167	1.632699939	2.283377245	0.829818027
Early_vs_ElderlyEA	GSE2585_THYMIC_DC_VS_THYMIC_MACROPHAGE_UP	-3.14751507	0.075835919	4.865714595	0.589129302	6.533303109	6.161250077	0.759793888	2.07400094	3.375672772	0.828816552
Early_vs_ElderlyEA	GAUCHER_PBMC_YF_VAX_STAMARIL_UNKNOWN_AGE_10DY_DN	-3.14737342	1.357742814	3.256624875	0.925161003	0.179148729	3.486531813	0.617799097	0.962481172	2.179180113	0.985999176
Early_vs_ElderlyEA	GSE29164_CD8_TCELL_VS_CD8_TCELL_AND_IL12_TREATED_MELANOMA_DAY7_DN	-3.14670946	29.51561601	5.316794313	0.51611756	4.351649823	2.878991932	0.6042468	5.604719254	2.044835698	0.393989731
Early_vs_ElderlyEA	GSE11961_MEMORY_BCELL_DAY7_VS_GERMINAL_CENTER_BCELL_DAY40_UP	-3.14366614	1.186474484	4.779312446	0.971459215	38.00614211	6.853826028	0.58788182	12.83043016	3.905823555	0.5096982
Early_vs_ElderlyEA	GSE17721_PAM3CSK4_VS_CPG_4H_BMDC_UP	-3.14291172	0.662230432	2.927700757	0.887945424	6.328525845	3.131673823	0.548245989	3.964279928	2.02419734	0.491655181
Early_vs_ElderlyEA	GSE1925_CTRL_VS_24H_IFNG_STIM_IFNG_PRIMED_MACROPHAGE_UP	-3.14154766	2.555078534	3.389253357	0.781361612	0.197415658	4.127591822	0.69189521	2.162723525	2.560901525	0.762860169
Early_vs_ElderlyEA	GSE17721_LPS_VS_POLYIC_16H_BMDC_DN	-3.14076964	0.375362845	4.101351945	0.810869244	32.12037571	4.008793455	0.38487783	7.285732879	2.63066666	0.449923374
Early_vs_ElderlyEA	GSE24634_IL4_VS_CTRL_TREATED_NAIVE_CD4_TCELL_DAY5_DN	-3.14076283	0.408701614	2.989580192	0.763979987	0.820361964	4.443817472	0.96445505	0.891836738	2.625712589	0.965224541
Early_vs_ElderlyEA	GSE43955_TH0_VS_TGFB_IL6_IL23_TH17_ACT_CD4_TCELL_60H_DN	-3.14059832	0.148713358	4.021298208	0.633418559	3.822054756	3.75718179	0.719214944	1.497996686	2.422611462	0.867448621
Early_vs_ElderlyEA	GOBP_MUSCLE_ADAPTATION	2.67566541	0.054217773	3.820707936	0.432961789	0.038989731	4.245459844	0.423397257	0.149365692	2.323390135	0.405673249
Early_vs_ElderlyEA	BERTUCCI_INVASIVE_CARCINOMA_DUCTAL_VS_LOBULAR_UP	2.78384039	0.465188944	3.618667963	0.83225975	12.48063675	2.746970979	0.336598847	6.176349835	1.865180226	0.320910209
Early_vs_ElderlyEA	WANG_NFKB_TARGETS	2.78623237	0.210106453	1.907684858	0.401944526	4.324630494	2.613312457	0.568468012	1.019409472	1.424264582	0.989231123
Early_vs_ElderlyEA	GOBP_NEGATIVE_REGULATION_OF_MUSCLE_CONTRACTION	2.79688925	0.180530748	2.936653174	0.555245977	0.00928947	4.816684492	0.287525476	0.064293837	2.435423053	0.243720865
Early_vs_ElderlyEA	BHATTACHARYA_EMBRYONIC_STEM_CELL	2.8207294	16.78551595	3.416847316	0.393073846	1.254388295	3.19126794	0.943369805	8.105463066	2.089093708	0.307532821
Early_vs_ElderlyEA	GOBP_ENDOPLASMIC_RETICULUM_ORGANIZATION	2.8215576	0.463688367	2.888211073	0.789682109	0.558777007	2.611136577	0.823054318	1.064122907	1.627635003	0.969539397
Early_vs_ElderlyEA	GOBP_CELL_COMMUNICATION_BY_ELECTRICAL_COUPLING_INVOLVED_IN_CARDIAC_CONDUCTION	2.8381546	0.000904258	5.889696806	0.175027723	0.061018159	3.092598564	0.345739887	0.075304164	1.932410926	0.170047572
Early_vs_ElderlyEA	GOBP_REGULATION_OF_MUSCLE_ADAPTATION	2.85197902	0.063028467	3.541172865	0.422538516	0.002014937	6.280110865	0.268959748	0.119749204	2.368209278	0.360149221
Early_vs_ElderlyEA	SWEET_KRAS_ONCOGENIC_SIGNATURE	2.88605119	0.622044613	2.939914343	0.871613394	9.180565055	3.061391396	0.455971515	5.585734314	2.04107935	0.392053136
Early_vs_ElderlyEA	KAAB_FAILED_HEART_ATRIUM_UP	2.92120812	2.572638327	2.416790045	0.693698328	6.222228864	7.417123392	0.805227571	7.938585968	2.517420412	0.404739444
Early_vs_ElderlyEA	GOBP_STRIATED_MUSCLE_CELL_DEVELOPMENT	2.95037978	0.32546758	1.880506774	0.545491479	0.183853533	2.593984789	0.502179477	0.364173498	1.494116955	0.49450015
Early_vs_ElderlyEA	GOBP_CELL_COMMUNICATION_INVOLVED_IN_CARDIAC_CONDUCTION	2.97290248	0.044184642	3.255565367	0.320365396	0.043275887	3.604173157	0.368058813	0.110038153	2.141867018	0.2949873
Early_vs_ElderlyEA	GOBP_POSITIVE_REGULATION_OF_CALCIUM_ION_TRANSMEMBRANE_TRANSPORTER_ACTIVITY	2.98890847	0.368361669	2.337964095	0.666540493	0.047368608	3.944805041	0.417854452	0.380974335	1.863718052	0.602074959
Early_vs_ElderlyEA	GOBP_CYTOPLASMIC_MICROTUBULE_ORGANIZATION	3.00979683	0.002656508	8.469050767	0.479281349	41.46696812	4.688263465	0.419851238	13.86975053	3.109761983	0.395985426
Early_vs_ElderlyEA	MARIADASON_REGULATED_BY_HISTONE_ACETYLATION_DN	3.04512271	0.070955428	4.223152452	0.524003635	289.7314888	5.761803403	0.295259048	7.775820945	2.86345935	0.47030748
Comparisons	Names	NES	AA.HR	AA.SE	AA.P-value	EA.HR	EA.SE	EA.P-value	All.HR	All.SE	All.P-value
EA_vs_AA	GOBP_REGULATION_OF_PROTEOLYSIS	-1.57283115	1.448628707	2.472460122	0.880802381	6.788863583	3.495281288	0.582034267	2.52355489	1.987288799	0.640852243
EA_vs_AA	GOBP_POSITIVE_REGULATION_OF_HYDROLASE_ACTIVITY	-1.57135098	0.83575295	2.44081622	0.94139703	21.95466649	3.414120273	0.356861953	2.066943057	1.888740006	0.700357278
EA_vs_AA	GOBP_POSITIVE_REGULATION_OF_PROTEOLYSIS	-1.57084159	3.264334663	2.228520033	0.593894789	103.6304182	3.527822763	0.179024802	10.52366032	1.879734926	0.20537658
EA_vs_AA	GOBP_MICROTUBULE_BASED_MOVEMENT	-1.55354452	16.87989867	2.321209297	0.215019387	250.3598437	3.630700991	0.126374052	50.69109763	1.921268402	0.036562338
EA_vs_AA	GOBP_REGULATION_OF_PEPTIDASE_ACTIVITY	-1.54748408	0.248220316	2.926068425	0.633033909	1.894936196	2.958879125	0.828807122	0.662482249	1.910349796	0.829289367
EA_vs_AA	GOBP_REGULATION_OF_MICROTUBULE_BASED_PROCESS	-1.53639243	2.271463769	2.072732574	0.691730771	64.8912989	3.373196678	0.203269506	8.580989761	1.787585232	0.225073603
EA_vs_AA	AFFAR_YY1_TARGETS_DN	-1.53321437	1.49818902	2.101276131	0.847391717	85.25085576	3.030407476	0.133706814	6.465654383	1.715581688	0.274324058
EA_vs_AA	GOBP_DEVELOPMENTAL_MATURATION	-1.52815517	0.28700505	2.788692796	0.653690936	3.977466421	3.249428609	0.669612954	0.707333926	1.984669439	0.861467691
EA_vs_AA	GOBP_ANATOMICAL_STRUCTURE_MATURATION	-1.5197955	0.369910932	2.761269397	0.718296895	4.34979312	3.198113617	0.644017796	0.80709292	1.973692894	0.913522035
EA_vs_AA	GSE18281_SUBCAPSULAR_CORTICAL_REGION_VS_WHOLE_CORTEX_THYMUS_DN	-1.50390359	3.827270702	2.324106777	0.561698731	1.952597483	3.236600987	0.836097978	3.774611548	1.892747511	0.481437486
EA_vs_AA	GOBP_REGULATION_OF_REPRODUCTIVE_PROCESS	-1.50366356	0.376908987	3.233613747	0.762749336	3.075365547	3.355697905	0.737464165	1.17552088	2.243516984	0.942537857
EA_vs_AA	GOBP_POSITIVE_REGULATION_OF_PEPTIDASE_ACTIVITY	-1.50160911	1.121452421	2.711585414	0.966280864	17.0346511	2.846044042	0.312030746	3.710823359	1.850520343	0.47728514
EA_vs_AA	GSE360_DC_VS_MAC_L_MAJOR_DN	-1.50132802	0.816240438	2.157415896	0.925008893	3.119466013	3.360085644	0.734421768	1.113034714	1.808485704	0.952779354
EA_vs_AA	PURBEY_TARGETS_OF_CTBP1_AND_SATB1_DN	-1.50116151	1.605014346	1.892706153	0.802453759	24.8087031	2.791292898	0.240209587	4.314023098	1.543043632	0.340662576
EA_vs_AA	GSE37301_COMMON_LYMPHOID_PROGENITOR_VS_GRAN_MONO_PROGENITOR_UP	-1.50053531	3.928911335	1.50544506	0.357192462	13.65116477	2.489155236	0.281730576	6.524038552	1.285912885	0.138160023
EA_vs_AA	REACTOME_CELL_SURFACE_INTERACTIONS_AT_THE_VASCULAR_WALL	1.43609239	0.280407832	1.775860035	0.47136815	4.334030983	1.744903051	0.393024898	0.956477487	1.117656149	0.968241297
EA_vs_AA	GOBP_ADAPTIVE_IMMUNE_RESPONSE	1.49476664	0.376689132	3.473508614	0.778616833	3.500005855	3.4352951	0.714771591	1.038791398	2.295645277	0.986773015
Comparisons	Names	NES	Elderly_EA.HR	Elderly_EA.SE	Elderly_EA.P-valu	Elderly_AA.HR	Elderly_AA.SE	Elderly_AA.P-valu	Total_Elderly.HR	Total_Elderly.SE	Total_Elderly.P-value
ElderlyAA_vs_EA	HALLMARK_ADIPOGENESIS	-1.22821463	3.286872963	1.262614216	0.34022972	2.785764654	1.587320364	0.514596851	3.627145875	0.985087727	0.184474187
ElderlyAA_vs_EA	GSE43955_TH0_VS_TGFB_IL6_IL23_TH17_ACT_CD4_TCELL_60H_DN	-1.22990797	1.255507527	1.614062659	0.887860076	1.497996686	2.422611462	0.867448621	1.370453842	1.343663302	0.81449749
ElderlyAA_vs_EA	GOBP_RIBONUCLEOSIDE_MONOPHOSPHATE_CATABOLIC_PROCESS	-1.69652263	1.024963423	1.295855043	0.984819085	0.339912676	1.555400038	0.484972363	0.60184092	0.9469299	0.591235032
ElderlyAA_vs_EA	BLANCO_MELO_COVID19_SARS_COV_2_LOW_MOI_INFECTION_A594_ACE2_EXPRESSING_CELLS_DN	-1.73691089	0.636139484	0.899317876	0.613788659	0.678407105	1.13897368	0.73265665	0.617723361	0.685357586	0.480348392
ElderlyAA_vs_EA	KEGG_PROXIMAL_TUBULE_BICARBONATE_RECLAMATION	1.65559919	1.248746369	1.586748779	0.888635449	2.771288574	1.91530523	0.592663237	1.8759311	1.223803535	0.606571057
ElderlyAA_vs_EA	GOBP_DEOXYRIBONUCLEOSIDE_MONOPHOSPHATE_METABOLIC_PROCESS	-1.62489274	0.951555054	1.703907452	0.976749961	1.032959302	1.797478731	0.985606297	1.03467662	1.170437543	0.976764811
ElderlyAA_vs_EA	GOBP_DEOXYRIBONUCLEOSIDE_METABOLIC_PROCESS	-1.754201	0.783679218	1.251530887	0.845530627	7.82776134	1.650277775	0.202757119	1.511691409	0.965436855	0.668387445
ElderlyAA_vs_EA	GOBP_NUCLEOBASE_CATABOLIC_PROCESS	-1.75419286	0.367293543	1.365748518	0.460284608	0.433321344	1.587893869	0.596819992	0.377645231	1.006761614	0.330594827
ElderlyAA_vs_EA	GOBP_PURINE_DEOXYRIBONUCLEOTIDE_CATABOLIC_PROCESS	-1.75420888	0.862452717	1.352151521	0.912847621	1.40044342	1.629042311	0.836078194	0.91666548	1.020608068	0.93205522
ElderlyAA_vs_EA	GSE17721_PAM3CSK4_VS_GADIQUIMOD_4H_BMDC_UP	-1.22806019	1.703891026	1.914619607	0.780518218	17.41869921	2.994238484	0.328726793	4.172674244	1.603727991	0.370050278
ElderlyAA_vs_EA	GOBP_GMP_METABOLIC_PROCESS	-1.52631178	4.947590941	1.475456269	0.271953556	13.20876626	1.855251125	0.139588875	7.241167865	1.1291771	0.071270965

Supplementary Table legends

Table S1 Demographics for samples analyzed for DNA methylation and RNA sequencing.

Table S2 Demographics for overlapping samples analyzed by both DNA methylation and RNA sequencing.

Table S3 Demographics of clinical samples included in the TMA.

Table S4 Percent AR positive nuclei in prostate tissues from AA and EA men.

Table S5 Differentially expressed nodes after AR KO between AA and EA men.

Table S6 Hazard ratios for GSEA pathways.
