# Supplementary material for: Race-specific coregulatory and transcriptomic profiles associated with DNA methylation and androgen receptor in prostate cancer
Source: Genome Med. 2024 Apr 2;16:52. doi: 10.1186/s13073-024-01323-6 (PMC10988846; doi:10.1186/s13073-024-01323-6)
Supplement: Supplementary file 2 — Additional File 2. [file 13073_2024_1323_MOESM2_ESM.pdf]

Figure S1

A.

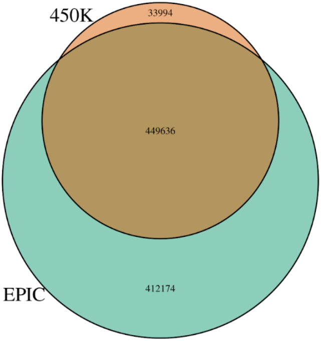

B.

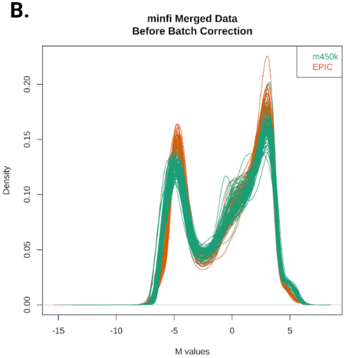

C.

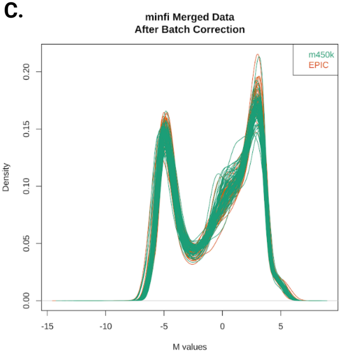

Figure S2

A.

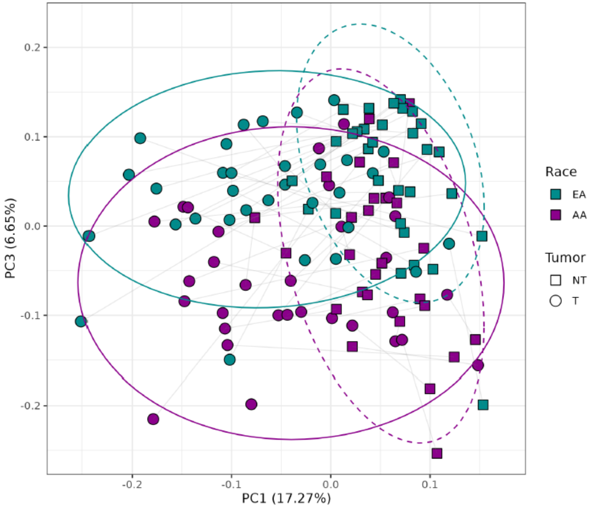

0.2
0.1
0.0
-0.1
-0.2
PC3 (6.65%)
-0.2
-0.1
0.0
0.1
PC1 (17.27%)
Race
EA
AA
Tumor
NT
T

B.

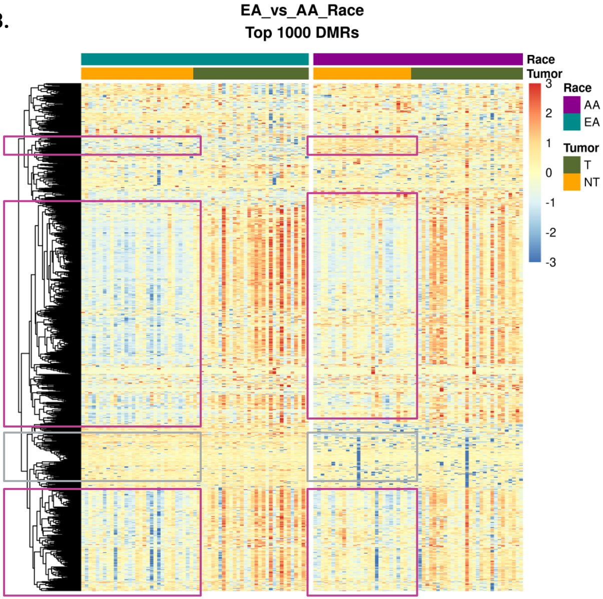

EA_vs_AA_Race
Top 1000 DMRs
Race
Tumor
3
2
1
0
-1
-2
-3
Race
AA
EA
Tumor
T
NT

Figure S3

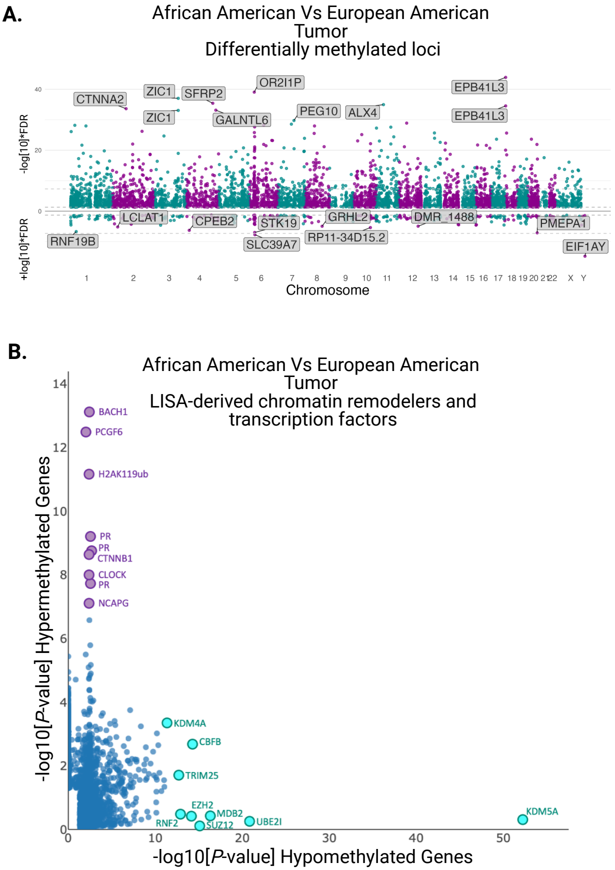
A.
African American Vs European American
Tumor
Differentially methylated loci
-log[10]*FDR
+log[10]*FDR
0
20
40
CTNNA2
ZIC1
ZIC1
SFRP2
GALNTL6
OR2I1P
PEG10
ALX4
EPB41L3
EPB41L3
LCLAT1
CPEB2
STK19
SLC39A7
GRHL2
RP11-34D15.2
DMR_1488
PMEPA1
EIF1AY
RNF19B
1
2
3
4
5
6
7
8
9
10
11
12
13
14
15
16
17
18
19
20
21
22
X
Y
Chromosome
B.
African American Vs European American
Tumor
LISA-derived chromatin remodelers and
transcription factors
BACH1
PCGF6
H2AK119ub
PR
PR
CTNNB1
CLOCK
PR
NCAPG
KDM4A
CBFB
TRIM25
EZH2
MDB2
RNF2
SUZ12
UBE2I
KDM5A
-log10[P-value] Hypermethylated Genes
-log10[P-value] Hypomethylated Genes
0
2
4
6
8
10
12
14
0
10
20
30
40
50

Figure S4

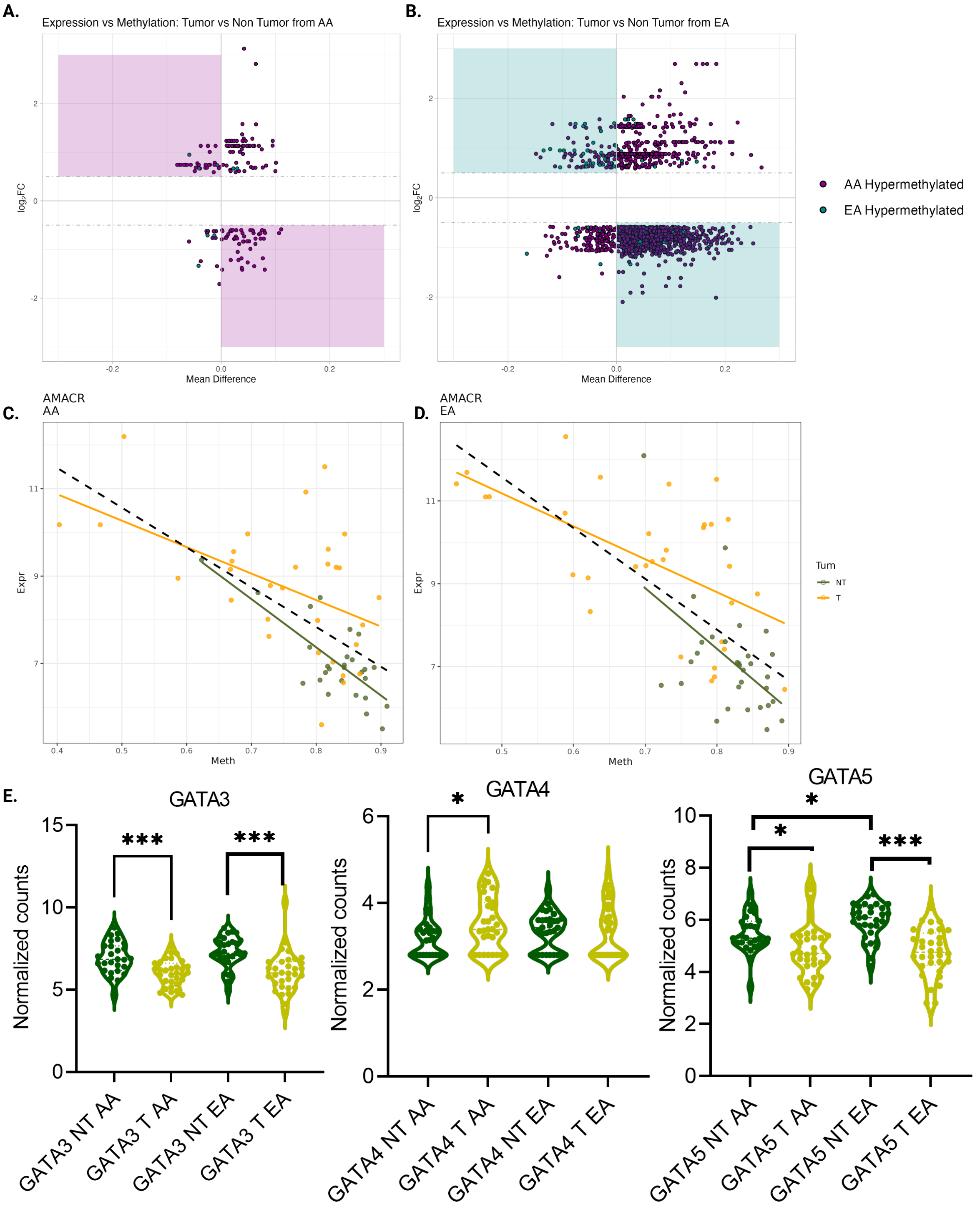
A.
Expression vs Methylation: Tumor vs Non Tumor from AA
log2FC
Mean Difference
B.
Expression vs Methylation: Tumor vs Non Tumor from EA
log2FC
Mean Difference
AA Hypermethylated
EA Hypermethylated
C.
AMACR
AA
Expr
Meth
D.
AMACR
EA
Expr
Meth
Tum
NT
T
E.
GATA3
Normalized counts
***
***
GATA3 NT AA
GATA3 T AA
GATA3 NT EA
GATA3 T EA
GATA4
*
Normalized counts
GATA4 NT AA
GATA4 T AA
GATA4 NT EA
GATA4 T EA
GATA5
*
*
***
Normalized counts
GATA5 NT AA
GATA5 T AA
GATA5 NT EA
GATA5 T EA

Figure S5

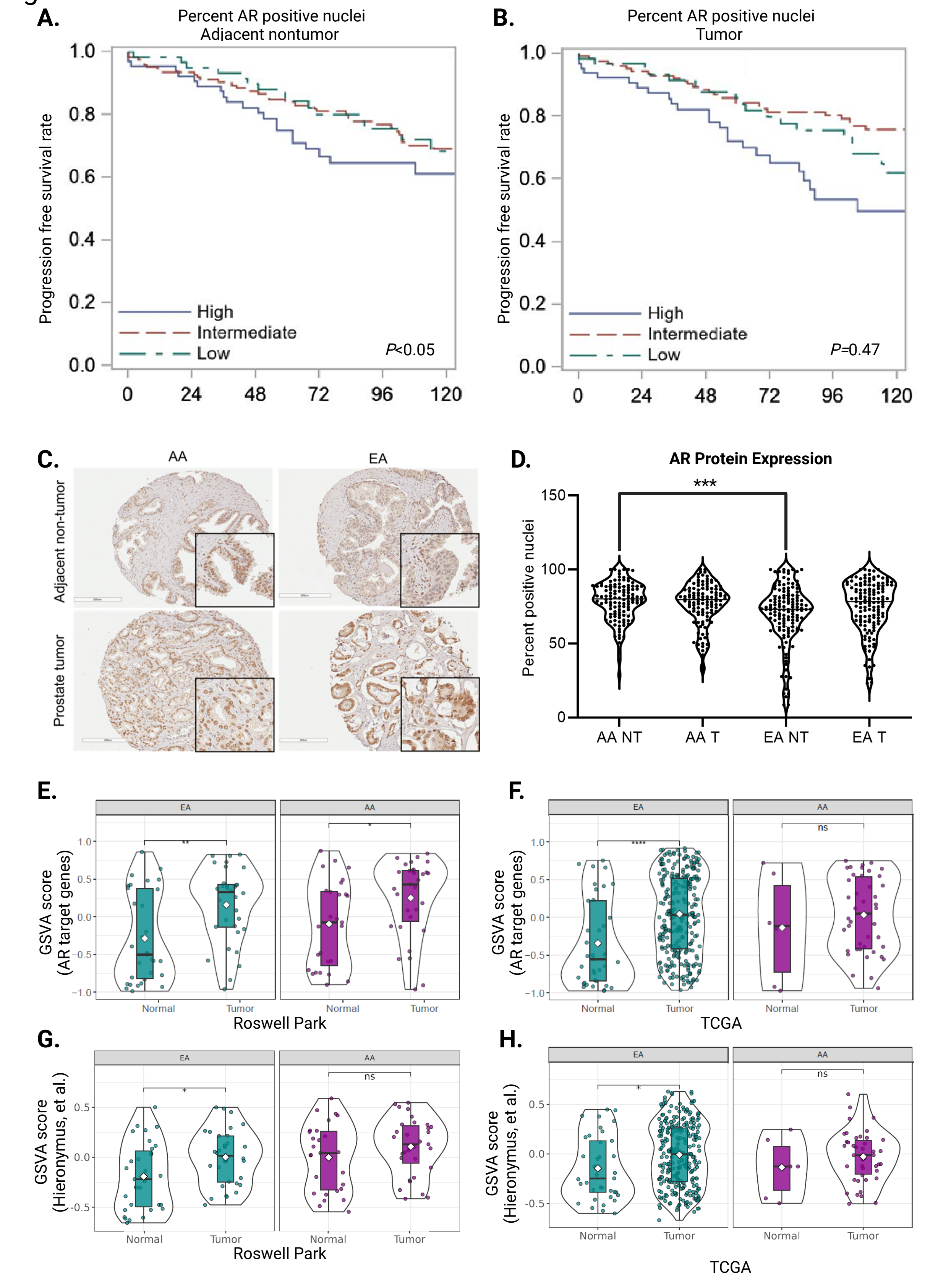

A.
Percent AR positive nuclei
Adjacent nontumor
Progression free survival rate
High
Intermediate
Low
P<0.05
B.
Percent AR positive nuclei
Tumor
Progression free survival rate
High
Intermediate
Low
P=0.47
C.
AA
EA
Adjacent non-tumor
Prostate tumor
D.
AR Protein Expression
Percent positive nuclei
***
AA NT
AA T
EA NT
EA T
E.
EA
AA
GSVA score
(AR target genes)
Normal
Tumor
Roswell Park
F.
EA
AA
GSVA score
(AR target genes)
ns
Normal
Tumor
TCGA
G.
EA
AA
GSVA score
(Hieronymus, et al.)
ns
Normal
Tumor
Roswell Park
H.
EA
AA
GSVA score
(Hieronymus, et al.)
ns
Normal
Tumor
TCGA

Figure S6

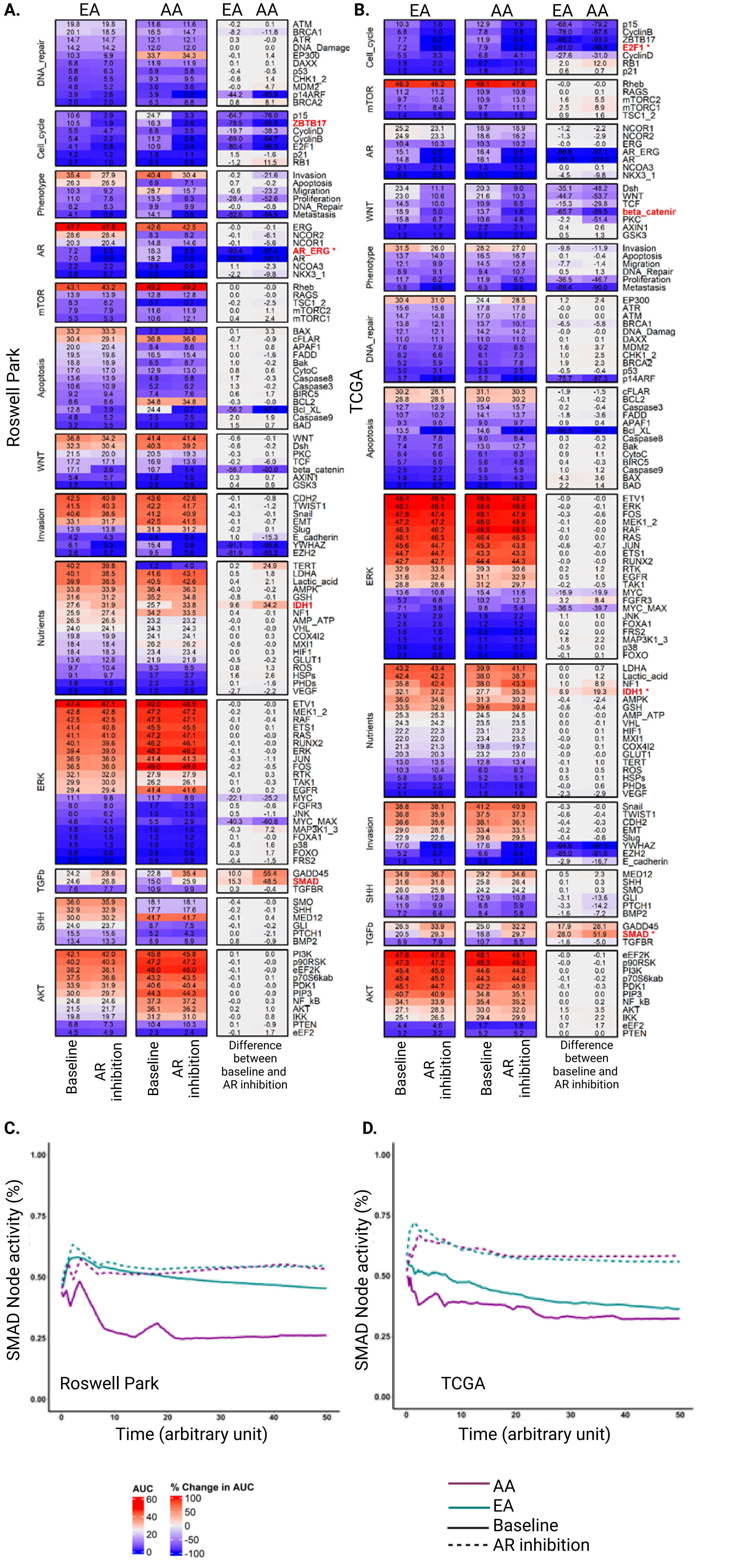
A.
B.
Roswell Park
TCGA
EA
AA
EA AA
Baseline
AR inhibition
Difference between baseline and AR inhibition
DNA_repair
Cell_cycle
Phenotype
AR
mTOR
Apoptosis
WNT
Invasion
Nutrients
ERK
TGFb
SHH
AKT
ZBTB17
AR_ERG *
IDH1
SMAD
E2F1 *
beta_catenin
IDH1 *
SMAD *
C.
D.
SMAD Node activity (%)
Time (arbitrary unit)
Roswell Park
TCGA
AUC
% Change in AUC
AA
EA
Baseline
AR inhibition

Figure S7

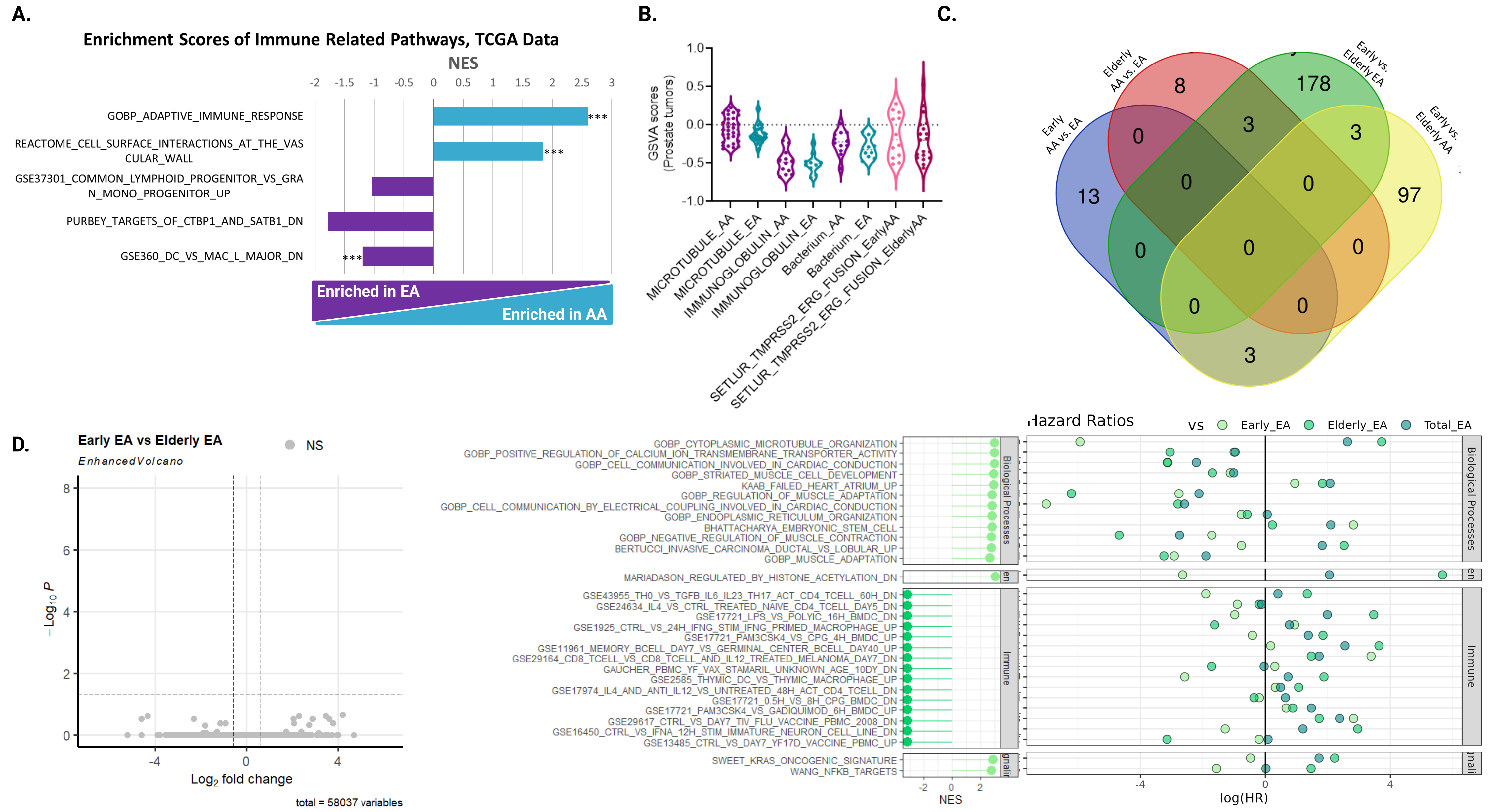
A.
Enrichment Scores of Immune Related Pathways, TCGA Data
NES
-2 -1.5 -1 -0.5 0 0.5 1 1.5 2 2.5 3
GOBP_ADAPTIVE_IMMUNE_RESPONSE
REACTOME_CELL_SURFACE_INTERACTIONS_AT_THE_VASCULAR_WALL
GSE37301_COMMON_LYMPHOID_PROGENITOR_VS_GRAN_MONO_PROGENITOR_UP
PURBEY_TARGETS_OF_CTBP1_AND_SATB1_DN
GSE360_DC_VS_MAC_L_MAJOR_DN
***
Enriched in EA
Enriched in AA
B.
GSVA scores (Prostate tumors)
1.0 0.5 0.0 -0.5 -1.0
MICROTUBULE_AA
MICROTUBULE_EA
IMMUNOGLOBULIN_AA
IMMUNOGLOBULIN_EA
Bacterium_AA
Bacterium_EA
SETLUR_TMPRSS2_ERG_FUSION_EarlyAA
SETLUR_TMPRSS2_ERG_FUSION_ElderlyAA
C.
Early AA vs. EA
Elderly AA vs. EA
Early vs. Elderly EA
Early vs. Elderly AA
13 8 178 97 0 3 3 0 0 0 0 0 0 0 3
D.
Early EA vs Elderly EA
EnhancedVolcano
NS
$-Log_{10}$ P
Log_2 fold change
total = 58037 variables
Hazard Ratios
vs Early_EA Elderly_EA Total_EA
GOBP_CYTOPLASMIC_MICROTUBULE_ORGANIZATION
GOBP_POSITIVE_REGULATION_OF_CALCIUM_ION_TRANSMEMBRANE_TRANSPORTER_ACTIVITY
GOBP_CELL_COMMUNICATION_INVOLVED_IN_CARDIAC_CONDUCTION
GOBP_STRIATED_MUSCLE_CELL_DEVELOPMENT
KAAB_FAILED_HEART_ATRIUM_UP
GOBP_REGULATION_OF_MUSCLE_ADAPTATION
GOBP_CELL_COMMUNICATION_BY_ELECTRICAL_COUPLING_INVOLVED_IN_CARDIAC_CONDUCTION
GOBP_ENDOPLASMIC_RETICULUM_ORGANIZATION
BHATTACHARYA_EMBRYONIC_STEM_CELL
GOBP_NEGATIVE_REGULATION_OF_MUSCLE_CONTRACTION
BERTUCCI_INVASIVE_CARCINOMA_DUCTAL_VS_LOBULAR_UP
GOBP_MUSCLE_ADAPTATION
MARIADASON_REGULATED_BY_HISTONE_ACETYLATION_DN
GSE43955_TH0_VS_TGFB_IL6_IL23_TH17_ACT_CD4_TCELL_60H_DN
GSE24634_IL4_VS_CTRL_TREATED_NAIVE_CD4_TCELL_DAY5_DN
GSE17721_LPS_VS_POLYIC_16H_BMDC_DN
GSE1925_CTRL_VS_24H_IFNG_STIM_IFNG_PRIMED_MACROPHAGE_UP
GSE17721_PAM3CSK4_VS_CPG_4H_BMDC_UP
GSE11961_MEMORY_BCELL_DAY7_VS_GERMINAL_CENTER_BCELL_DAY40_UP
GSE29164_CD8_TCELL_VS_CD8_TCELL_AND_IL12_TREATED_MELANOMA_DAY7_DN
GAUCHER_PBMC_YF_VAX_STAMARIL_UNKNOWN_AGE_10DY_DN
GSE2585_THYMIC_DC_VS_THYMIC_MACROPHAGE_UP
GSE17974_IL4_AND_ANTI_IL12_VS_UNTREATED_48H_ACT_CD4_TCELL_DN
GSE17721_0.5H_VS_8H_CPG_BMDC_DN
GSE17721_PAM3CSK4_VS_GADIQUIMOD_6H_BMDC_UP
GSE29617_CTRL_VS_DAY7_TIV_FLU_VACCINE_PBMC_2008_DN
GSE16450_CTRL_VS_IFNA_12H_STIM_IMMATURE_NEURON_CELL_LINE_DN
GSE13485_CTRL_VS_DAY7_YF17D_VACCINE_PBMC_UP
SWEET_KRAS_ONCOGENIC_SIGNATURE
WANG_NFKB_TARGETS
Biological Processes
Immune
NES
log(HR)

Supplementary Figure Legends

Figure S1 DNA methylation data obtained from 450K and EPIC arrays have large overlap and concordance. A. Venn diagram depicting the number of probes on the 450K DNA methylation array (orange circle, n=483,630), the EPIC DNA methylation array (green circle, 861,810), and the number of probes overlapping between both arrays which were the only probes considered for downstream analysis (449,636). **B.** Density plot of M values output from minfi for both 450K and EPIC arrays prior to batch correction. **C**. Density plot of M values output from minfi for both 450K and EPIC arrays after batch correction, demonstrating a loss of batch effect.

Figure S2 Race and tumor vs. non-tumor comparison of differentially methylated regions. A. Principal component analysis (PCA) of DNA methylation profiles from prostate tumors and adjacent non-tumor tissue from AA and EA men. PCA plots show that tissues from AA and EA men are distinct, but there is no obvious separation between prostate cancer and adjacent non-tumor tissue. Solid lines represent 95% confidence ellipses for tumor samples, grouped by race (AA: Purple, EA: Blue) and dashed lines represent 95% confidence ellipses for no-tumor samples based on race (AA: Purple, EA: Blue) **B.** Heatmap representing top1000 differentially methylated regions in prostate tumors and adjacent non-tumor tissues from AA and EA men. Pink boxes represent groups of DMRs with increased DNA methylation in non-tumor tissues of AA samples compared to EA, and gray boxes represent groups of DMRs with decreased DNA methylation in non-tumor tissues of AA compared to EA samples.

Figure S3 Differentially methylated regions between prostate tumors from AA and EA men reveal race-specific enrichment of pathways and transcript factors. A. Manhattan plots representing differentially methylated regions in prostate tumors from AA and EA men across all

chromosome locations. **B.** Lisa-derived epigenetic regulators with binding enrichment in hypermethylated or hypomethylated genes in prostate tumors from AA compared to EA men.

Figure S4 The relationship between DNA methylation and gene expression correlation are distinct in a subset of genes from prostate tumors between AA and EA men. A. Comparisons between prostate tumors and adjacent non-tumor tissues of AA men. **B.** Comparisons between prostate tumors and adjacent non-tumor tissues of EA men. Each dot represents ρ, correlation coefficient, between differentially methylated regions (DMRs) and gene expression. *X-axis* represents the mean difference in DMRs, and *Y-axis* represents log fold change in gene expression. The shaded areas represent an inverse correlation between DNA methylation and gene expression. **C-D.** Negative correlation is observed between DNA methylation and AMACR gene expression in prostate cancer and adjacent non-tumor tissue from AA men (*left* panel, **C**) and EA men (*right* panel, **D**). *Y-axis* represents gene expression and *X-axis* represents DNA methylation. Each dot is the correlation estimate in each individual sample. **E.** Normalized counts from RNA-sequencing data of the GATA family members in tissues from AA and EA men with prostate cancer.

Figure S5 Tumor AR transcriptional activity is upregulated when compared to normal prostate tissue despite similar nuclear AR protein expression in both. A-B. Progression free survival was significantly worse in men with a higher percent of AR positive nuclei in adjacent non-tumor tissues (**A**) but not prostate tumors (**B**). **C.** Representative images of AR expression in prostate tumors and adjacent non-tumor tissues from AA and EA men. **D**. Violin plots show significantly higher AR-positive nuclei in adjacent non-tumor tissues from AA men than EA men. Each dot represents the average percent of positive AR nuclei in triplicate tissue cores. NT: Adjacent non-tumor tissue and T: Prostate tumors. Number of samples: AA = 107, EA = 133; ****P* <0.001. **E.-F.** GSVA scores were calculated for AR transcriptional targets: *KLK2*, *KLK3*,

TMPRSS2, and *NKX3.1*. AR transcriptional activity calculated for normal (non-tumor) and tumor tissues of EA (left panel) and AA prostate tumors (right panel) are represented in the violin for the Roswell Park cohort **(E)** and the TCGA cohort (**F**) **G.-H** GSVA scores calculated for a 27-gene signature to represent AR transcriptional activity (Hieronymus et al., (2006)). AR transcriptional activity scores calculated from this gene signature for normal (non-tumor) and tumor tissues of EA (left) and AA (right) are represented in the violin for the Roswell Park cohort (**G)** and the TCGA cohort (**H**). Each dot represents a single sample; ns is non-significant. Each dot represents a single tissue. **P*<0.05, ***P*<0.01, ****P*<0.001.

Figure S6 Simulation of AR inhibition in prostate cancer-specific Boolean network reveals significant changes in AR-associated transcriptional programs in prostate cancer from AA men. A. and B. Changes in all the pathways included in the Boolean network before and after simulation of AR inhibition between race are represented in the heatmap for Roswell Park and TCGA cohort. *A*: Roswell Park clinical samples, *B*: TCGA clinical samples. Percent change in SMAD AUC (highlighted in red) was significantly different between prostate tumors from AA and EA men in both RP and TCGA clinical samples. Changes in AUC are represented by blue (down) and red (up). **C and D.** Changes in *SMAD* expression over an arbitrary period of time are represented in the graphs. Purple line: median of all prostate tumors from AA men. Blue line: median of all prostate tumors from EA men. Solid line: before AR inhibition and dashed line: simulation of AR inhibition. Solid line: before AR inhibition and dashed line: simulation of AR inhibition. *Left panel*: Roswell Park cohort, *Right panel*: TCGA cohort.

Figure S7 Race comparisons reveal distinct gene enrichments that correlate with progression-free survival in early-onset disease. A. Gene set enrichment analysis of differentially expressed genes between EA and AA patients from the TCGA cohort. **B.** Age-

specific comparisons show the common and distinct pathways in prostate tumors. **C.** Number of differentially expressed genes for each comparison (Early AA vs. EA, Elderly EA vs. AA, Early vs. Elderly EA, Early vs. Elderly AA) and number of genes which overlapped between the four comparisons. **D.** Comparisons of prostate tumors from EA men younger than 55 and EA men >=55 years. *Left* panel represent differentially expressed genes utilizing DESeq2, *Middle* panel represent gene set enrichment analysis based on clusterProfiler, and R*ight* panel represent hazard ratios based on calculated GSVA scores.
